# From the Mountain to the Valley: Drivers of Groundwater Prokaryotic Communities along an Alpine River Corridor

**DOI:** 10.3390/microorganisms11030779

**Published:** 2023-03-17

**Authors:** Alice Retter, Johannes Christoph Haas, Steffen Birk, Christine Stumpp, Bela Hausmann, Christian Griebler, Clemens Karwautz

**Affiliations:** 1 Department of Functional and Evolutionary Ecology, University of Vienna, 1030 Wien, Austria; 2 Institute of Earth Sciences, NAWI Graz Geocenter, University of Graz, 8010 Graz, Austria; 3 Institute of Soil Physics and Rural Water Management, University of Natural Resources and Life Sciences (BOKU), 1180 Wien, Austria; 4 Joint Microbiome Facility of the Medical University of Vienna and the University of Vienna, 1030 Wien, Austria; 5 Department of Laboratory Medicine, Medical University of Vienna, 1090 Wien, Austria

**Keywords:** aquifer, prokaryotic diversity, community assembly, aquatic ecology

## Abstract

Rivers are the “tip of the iceberg”, with the underlying groundwater being the unseen freshwater majority. Microbial community composition and the dynamics of shallow groundwater ecosystems are thus crucial, due to their potential impact on ecosystem processes and functioning. In early summer and late autumn, samples of river water from 14 stations and groundwater from 45 wells were analyzed along a 300 km transect of the Mur River valley, from the Austrian alps to the flats at the Slovenian border. The active and total prokaryotic communities were characterized using high-throughput gene amplicon sequencing. Key physico-chemical parameters and stress indicators were recorded. The dataset was used to challenge ecological concepts and assembly processes in shallow aquifers. The groundwater microbiome is analyzed regarding its composition, change with land use, and difference to the river. Community composition and species turnover differed significantly. At high altitudes, dispersal limitation was the main driver of groundwater community assembly, whereas in the lowland, homogeneous selection explained the larger share. Land use was a key determinant of the groundwater microbiome composition. The alpine region was more diverse and richer in prokaryotic taxa, with some early diverging archaeal lineages being highly abundant. This dataset shows a longitudinal change in prokaryotic communities that is dependent on regional differences affected by geomorphology and land use.

## 1. Introduction

Aquifers are of key importance for surface aquatic and terrestrial ecosystems, as well as for humans, in the form of drinking water, water for industrial uses, and water for irrigation. Invisible to the human eye, groundwater ecosystems are populated by diverse communities composed of microorganisms and metazoans well adapted to the darkness and energy scarcity that prevails in the subsurface [1,2,3,4]. While the minute interstices of unconsolidated sediments and the often narrow channels and cracks in karst and fissured aquifers may represent a spatial limitation for metazoans, microorganisms are not restricted by this and are distributed ubiquitously throughout the subsurface [3,5,6]. Because of their diverse and metabolically flexible lifestyles, they are closely linked to most element cycles, with the carbon, nitrogen, and phosphorus cycles being relevant examples [7,8,9]. Besides being involved in processes such as organic matter (OM) sequestration, degradation, and mineralization, microorganisms serve as a source of food for other members of the groundwater food web [10,11].

Groundwater ecosystems in alpine regions are of particular interest. On one hand, mountainous areas are considered an important freshwater resource and biodiversity hotspot [12,13]. On the other hand, alpine regions, as well as groundwater ecosystems are believed to be especially sensitive to external disturbances such as increased input of carbon, nutrients, pollutants, and heat [14,15,16,17]. Shallow aquifers are at the transition between highly dynamic surface environments and deep, stable groundwater systems. While already depleted in organic carbon and most nutrients, they may receive irregular pulses of energy along with hydrological events such as heavy precipitation and floods, as well as intensive land use activities, such as the application of mineral fertilizers and manure. Agricultural practices play an important role in the determination of the microbial community composition, as they often mobilize OM that is stably stored in the soil or alter hydrologic conditions in groundwater through irrigation [18,19]. The composition and functioning of microbial communities in groundwater are generally closely linked to dissolved organic matter (DOM) turnover, hydrochemical conditions, as well as aquifer lithology [20,21,22], but also by physical limitations, such as geographic distance [22,23]. Microbial community assembly, on the other hand, which determines the identity and abundance of taxa, is the result of stochastic and deterministic processes [24]. The contribution of deterministic environmental factors and stochastic, or random, processes such as dispersal or disturbance (for example, precipitation, drought, anthropogenic impacts) can be determined [25] and used for predictive models and management purposes [26,27].

Besides general influence from nearby surface waters such as rivers and streams, precipitation, and agricultural activities, urban areas constitute another prominent impactto groundwater ecosystems [28]. Groundwater below urban areas is highly impacted by surface sealing and reduced recharge, accumulation of heat, and the import of various kinds of contaminations including aromatic hydrocarbons, heavy metals, salts, and nanoparticles [29,30,31].

There is a suite of ecological concepts proposed to be valid in macroecology and microbial ecology, while validation in microbial settings lag behind [32,33,34]. Hardly any of these concepts have been tested systematically for their validity in groundwater ecosystems [2,35]. Three ecological concepts are tested here. The species–area relationship, which describes the relationship between species richness as a function of habitat size [36], is still pending evaluation for groundwater systems. However, one would assume that here too, species richness increases with aquifer volume and groundwater catchment size. The second ecological concept, i.e., the intermediate disturbance hypothesis, assumes that microbial phylogenetic and functional diversity peak at intermediate intensities or frequencies of disturbances [37,38]. Disturbances in aquifers can be the result of multiple concurrent stressors, including hydrological events and pollution [39]. The third concept relates to the input of energy into the groundwater. Here, we will test whether, according to the existing productivity–diversity concept [40], species diversity is highest at sites of moderate energy input, for example, in groundwater that is close to the surface or those that are hydrodynamically well connected to surface waters.

We examined physico-chemical and microbial characteristics, as well as drivers of microbial community composition and assembly in the groundwater, with respect to differences between groundwater and river water. To this end, shallow groundwaters were studied: along a gradient from mountainous to lowland areas; and from areas with natural vegetation to land impacted by agriculture and urban development. In addition, the ecological concepts mentioned above were tested with this dataset. More than 45 sites distributed along an alpine and pre-alpine transect of the River Mur (Austria) were sampled in early summer and autumn 2020.

We investigated the following questions:

(1) Do near-surface aquifers resemble adjacent surface waters, or are the environmental conditions (low energy, low temperatures, and darkness) and proximity to certain influencing factors such as land use or hydrological conditions determining community composition and assembly?
(2) What community assembly processes shape the prokaryotic community in groundwater, and do they differ from those in surface waters?
(3) Do patterns of groundwater microbial communities correspond to ecological concepts known from surface ecosystems in terms of biodiversity, energy, productivity, and disturbance?

The DNA-derived community is considered a species pool including dead or dormant cells that are negligible for the structure, functioning or response of the groundwater microbial communities at the time of sampling [41]. To avoid this bias, we answered the aforementioned questions in the context of the metabolically active (i.e., RNA derived) fraction of the prokaryotic community [42], where a comparison between the total (i.e., derived from the DNA) and the active prokaryotic community was made beforehand.

## 2. Materials and Methods

### 2.1. Field Work

The sampling of groundwater and surface water took place in June to July 2020 (early summer) and October to November 2020 (late autumn). Overall, 118 samples were collected in total, consisting of 90 groundwater samples (45 in early summer and late autumn each), and 28 surface water samples, i.e., 24 Mur River samples, and 4 samples from 2 tributaries (rivers Pöls and Lassnitz) (geographic coordinates see Table S1).

### 2.2. Study Area

**Figure 1:**
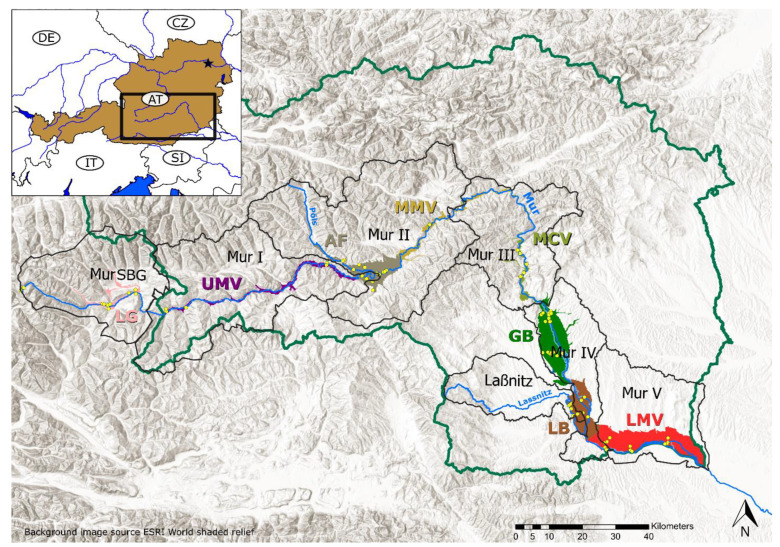
Groundwater, and surface water sampling sites (yellow points) along the Mur Valley in Styria (green boundaries) and Salzburg located along 8 distinct groundwater bodies that are named after their associated Mur Valley sub regions (color code, name (abbreviation) as follows: pink, Lungau (LG); violet, Upper Mur Valley (UMV); grey, Aichfeld (AF); ochre, Middle Mur Valley (MMV); olive, Mur Cross Valley (MCV); green, Graz Basin (GB); brown, Leibnitz Basin (LB); red, Lower Mur Valley (LMV)), as well as Mur River catchments sections (Mur SBG, Mur I, Mur II, Mur III, Mur IV, Laßnitz, Mur V). (Map redrawn from Haas & Birk [44].)

The sampled area is located along the Mur Valley in the provinces Salzburg and Styria, Austria (Figure 1), and it covers a longitudinal transect of 325 km and an elevation gradient of approx. 1600 m. The Mur River emerges from a karst spring at an elevation of 1898 m a.s.l. in the Hohe Tauern National Park, at the main chain of the alps, and flows downgradient through the alpine foreland to the lowlands at the border with Slovenia, where it leaves Austria at an elevation of about 260 m a.s.l. The transect combines 8 distinct groundwater bodies of different sizes, belonging to one large system of aquifers (Figure 1) that are, with the exception of the River Mur source, composed of alluvial and fluvio-glacial sediments, consisting of a mix of local and upstream lithologies. In the upper section from about 2000 m a.s.l. down to the city of Graz at 350 m a.s.l., the bedrock lithology embedding the shallow porous aquifer of the Mur Valley consists mostly of metamorphic units of the Austroalpine Nappe, covering a wide range of lithologies from metamorphic, silica-rich basement to Paleozoic limestones. Downstream of the city of Graz the underlying lithology is replaced by Neogene sediments which are also found in some alpine basins upstream such as the Aichfeld [43].

### 2.3. Samples Collection

Groundwater was withdrawn by a submersible pump (Grundfos MP1, Eijkelkamp Soil & Water, Giesbeek, Netherlands) from approx. 2 m below the water table in fully screened wells or otherwise from 2 m below the screened zone. Before sample collection, stagnant well water was replaced by pre-pumping twice the well water volume until reaching stability in key physico-chemical parameters (EC, pH, Temp, Dissolved Oxygen) [45]. Surface water samples were collected from the banks of the rivers Mur, Laßnitz, and Pöls, lowering all bottles and vials at least 10–20 cm below the water level.

### 2.4. Analyses of Physico-Chemical and Microbial Parameters

Water samples were analyzed for major ions and for nutrient concentrations, prokaryotic cell counts, microbial ATP, stable water isotopes signature (δ^18^O, δ^2^H), dissolved organic carbon (DOC), and dissolved inorganic carbon (DIC), as previously described in Retter et al. [46]. Groundwater and surface water samples for the measurement of microbial ATP, concentration of major ions, and stable water isotope ratios were filled into autoclaved glass bottles. For prokaryotic cell counts, samples were collected in sterile 15 mL plastic tubes and fixed with glutardialdehyde (0.5% *v*/*v*, final concentration) on site. Samples for DOC and DIC measurement were filtered through a 0.45 µm pre-rinsed PVDF syringe filter (STARLAB International, Hamburg, Germany) on site into acid-washed (5% HCl) glass vials. DOC samples were subsequently acidified with HCl to a pH ≤ 2. All samples were kept in the dark at 4–8 °C and analyzed within 48 h.

Water temperature, pH, EC, and concentration of dissolved oxygen (DO) of groundwater and river were measured on site using field sensors (WTW, Weilheim, Germany).

#### 2.4.1. Total Prokaryotic Cell Count

The total number of prokaryotic cells in water samples was quantified by means of flow cytometry (Amnis CellStream, Luminex, Austin, TX, USA) equipped with a 488 nm blue light laser. Settings of the different detector channels were as follows: forward scatter, side scatter, trigger channel laser (488 nm) were all at 100%; speed was ‘high’ (14.64 µL min^−1^). To distinguish intact prokaryotic cells from damaged cells or inorganic particles, prokaryotes were stained with nucleic acid dye SYBR Green I (Invitrogen, Darmstadt, Germany) at a volume ratio of 1:10,000 and incubated for 13 min at 37 °C. Cell counts were conducted in technical duplicates. Total cell counts (cells m L^−1^) were calculated using the Amnis CellStream Acquisition and Analysis software (v. 1.3.389).

#### 2.4.2. Determination of Microbial Intracellular ATP

Microbial intracellular ATP concentrations were determined using the BacTiter-Glo Microbial Cell Viability Assay (Promega, Madison, WI, USA) based on the protocol by Hammes et al. [47] with modifications as follows: The assay reagent (prepared following the manufacturer’s instructions) and samples were pre-warmed separately to 37 °C for 3 min before mixing 180 µL of sample with 20 µL of reagent, and subsequent incubation for 20 s at 37 °C while shaking at 600 rpm on a Thermomixer (Eppendorf). The luminescence signal was measured in a GloMax Navigator plate reader (Promega) with an integration time of 0.3 s. Concentrations were determined against ATP standards dissolved in ATP-free water. To correct for the contribution of extracellular ATP in the samples, each sample was measured twice, once untreated, and once after the sample was centrifuged for 30 min at 21,000× *g* and 4 °C resulting in a supernatant containing only extracellular ATP. The concentration of intracellular ATP was calculated by subtracting the concentration of extracellular from total ATP. Each sample was analyzed in technical triplicates.

#### 2.4.3. Determination of DOC, and DIC

Concentration of dissolved organic carbon (DOC) and dissolved inorganic carbon (DIC) in surface water and groundwater samples were measured in a TOC-L Analyzer (Shimadzu, Kyoto, Japan). DOC was quantified as non-purgeable carbon in acidified samples [48]. DIC was quantified from purged CO_2_ after acidification. Calculation of DOC and DIC concentration made use of pre-measured standard curves.

#### 2.4.4. Water Stable Isotope Signature

Water samples were analyzed for its ratios of ^18^O/^16^O and ^2^H/^1^H using laser spectroscopy (Picarro, L2140-i). All samples were referenced to the VSMOW-SLAP (Vienna Standard Mean Ocean Water-Standard Light Antarctic Precipitation) standard, and results are reported as the ratios of isotopes in the delta notation as δ-value (‰). Precision of the instrument (1σ) was better than 0.15‰ and 0.6‰ for δ^18^O and δ^2^H, respectively.

#### 2.4.5. Major Ions and Nutrients

Major ion concentrations (Na^+^, K^+^, Ca^2+^, Mg^2+^, Cl^−^, SO_4_^2−^) were measured by ion chromatography (Dionex ICS-1100 RFIC; Thermo Scientific, Idstein, Germany) following standard protocols (OENORM DIN EN ISO 14911, DIN EN ISO 10304-1). Nutrient concentrations (Table S2) were determined by photometric measurements.

#### 2.4.6. Microbial Community Analysis

For molecular analysis of Bacteria and Archaea, groundwater and river water samples were filled into sterilized (2% sodium hypochlorite solution) 10 L plastic containers. Within 48 h of sampling, in which containers were stored in a dark at 4–8 °C, a total of 10 L of groundwater and 4 L of surface water were filtered onto 0.22 µm Sterivex filters (Merck Millipore, Darmstadt, Germany) using a peristaltic pump. All tubing were pre-washed with sodium hypochlorite, rinsed with MilliQ and sample water before filtration. Filters were stored right away at −80 °C until further analysis.

### 2.5. Nucleic Acid Extraction and Illumina Sequencing

Genomic DNA and total RNA were extracted in two batches (early summer and late autumn samples), using the Norgen RNA/DNA purification kit (Norgen Biotek, ON, Canada), where nucleic acids are extracted and sequentially purified over two spin columns. Briefly, filters were first removed under sterile conditions and then cut to small pieces, and 800 µL of the kit’s lysis buffer was added to the filter strips in a sterile 1.5 mL screw cap micro tube containing 0.2 mL of a mix of 1:1 0.1 mm and 0.7 mm Zirconia/Silica beads (Biospec, Bartlesville, USA). Cell lysis was enhanced by incubation for 10 min at 55 °C, followed by bead-beating. The entire liquid was transferred to a new micro-centrifuge tube and centrifuged at 14,000× *g* for 2 min. The supernatant was then purified according to the manufacturer’s protocol and DNA and RNA was quantified using a Qubit 4 HS RNA, and HS dsDNA assay (Invitrogen). To digest undesired DNA within the RNA fraction, samples were treated with TURBO Dnase (2 Units/μL) (Thermo Fisher Scientific, Waltham, MA, USA), additionally adding RNaseOUT (Thermo Fisher Scientific, USA) to avoid RNA degradation during DNAse treatment, and cleaning up with the Norgen CleanAll DNA/RNA Clean-Up and Concentration Micro Kit to remove any downstream enzymatic reactions inhibitors (Norgen Biotek, Thorold, ON, Canada). The absence of DNA was checked by attempting the amplification of the 16S rRNA gene (35 amplification cycles), and TURBO Dnase treatment of individual samples was repeated if amplification occurred. For further downstream processing of the RNA samples, cDNA was synthesized with the RevertAid First Strand cDNA Synthesis kit (Thermo Fisher Scientific, USA) following the manufacturer’s protocol. A primer combination of 515F (5′-GTGYCAGCMGCCGCGGTAA-3′, [49]) and 806R (5′-GGACTACNVGGGTWTCTAAT-3′, [50]), annealing to the V4 region of the 16S rRNA was used for gene amplification, following the PCR protocol as described by Pjevac et al. [51]. Sequencing was performed on a MiSeq platform (Illumina, San Diego, CA, USA), using a v3, 600 cycle chemistry (2 × 300 b paired-end sequences), in two separate runs. Negative controls (*n* = 3) were extracted from a blank sterile 0.22 µm Sterivex filter (Merck Millipore, Darmstadt, Germany), following the same extraction protocol as the biological samples.

### 2.6. Sequence Data Processing

Raw reads were processed in R (version 4.1.1, [52]) with DADA2 (v. 1.20.0; [53]) as outlined in Callahan et al. [53]. Amplicon Sequence Variants (ASVs; [54]) were inferred across all samples in pooled mode (details on sequence trimming, and quality filtering is outlined in Pjevac et al. [51]).

### 2.7. Sequence Classification and Decontamination

ASV sequences were subsequently aligned and classified using SINA version 1.7.2 [55] and the SILVA database SSU Ref NR 99 release 138.1 [56] using default parameters. ASVs classified as eukaryotes, mitochondria, or chloroplasts, as well as ASVs not classified at domain level, were removed from the dataset prior to downstream analysis. One sample did not deliver any reads (groundwater sample 86). The dataset was bioinformatically decontaminated in R (v. 4.1.2, [52]) with decontam (v. 1.14.0, [57]), with a prevalence-based identification of contaminated sequences, using a strict threshold of 0.5, identifying all sequences as contaminants that are more prevalent in the negative controls (*n* = 3) than in the samples. Summer and autumn sequencing batches were analyzed separately against the respective negative control samples.

A species tree was estimated from Bacterial and Archaeal 16S rRNA-derived ASVs, adjusting read direction with MAFFT (v. 7.427; [58]), subsequent multiple sequence alignment with Clustal Omega (v. 1.2.4; [59]), and estimating the maximum likelihood tree with a GTR + F + ASC + R10 evolutionary model performing an SH-like approximate likelihood ratio test (SH-aLRT) [60] as well as ultrafast bootstrap [61] with 1000 replicates using iqtree (v. 2.0.3; [62]).

### 2.8. Classification of Hydrology, Land Use and Disturbances

The groundwater wells were grouped according to their probability of being influenced by surface water intrusion based on the minimum surface water infiltration distance (with categories: none, weak, moderate, high). This distance was derived from a map of groundwater level contour lines, drawn from the water table data of public wells, as well as from expert knowledge about the local hydrogeological conditions.

In order to link precipitation events and dynamics in river levels, we used the Standardized Precipitation Index (SPI, [63]), the Standardized Groundwater level Index (SGI, [64]), and the Standardized River Stages Index (SRSI, [44]). These so-called drought indices allowed different time series to be compared by standardizing them into a range of ±3 standard deviations. Additionally, the SPI allowed for the use of averaging periods (9–12 months in our case), taking into account that surface water and groundwater typically react to precipitation with some delay and attenuation such that short-term fluctuations are smoothed out. SPI and SRSI were correlated to SGI via a Pearson correlation, which were then categorized based on the resulting correlation coefficient into none (<0.3), weak (0.3–0.49), moderate (0.5–0.8), and strongly (>0.8) correlating wells.

Groundwater level fluctuations between the absolute long-term minimum and maximum measured for the time period 2000 to 2019 were used to categorize groundwater sites into low dynamic (average groundwater level fluctuation between 0 and 1.5 m), medium dynamic (average groundwater level fluctuation between 1.5 and 3 m), and highly dynamic (average groundwater level fluctuation between 3 and 7 m).

Moreover, information on land cover was taken from the Corine database (2012–2018; https://www.data.gv.at/, accessed on 1 February 2021). Surface water samples were manually classified as ‘water ways’.

Factors chosen to test the intermediate disturbance hypothesis are (1) levels of nitrate in groundwater, a proxy for intensive agricultural land use, (2) absolute difference in groundwater level between the long-term minimum and maximum, as well as (3) the proximity to the nearest river water infiltration point. To test the productivity–diversity concept, total prokaryotic cell count and intracellular ATP concentrations were used as proxies for the prokaryotic secondary production in the groundwater ecosystem.

### 2.9. Statistical Analysis

For ordination of samples based on environmental parameters, a principal component analysis (PCA) was conducted, where parameters were standardized to z-scores beforehand. The correlation between individual environmental parameters was evaluated via a Spearman correlation matrix. To test if environmental variables change between groups (e.g., groundwater and the river, or early summer and late autumn), a multiple Student’s t test was conducted, where *p*-values were false discovery rate (FDR) adjusted, and significance was given based on a cut-off of <0.05.

First, the analysis of the groundwater prokaryotic communities was based on DNA (total community) and RNA (active community) to compare both fractions in terms of their diversity and taxonomic composition. The subsequent analysis comparing groundwater to the river, as well as diversity within groundwater and its link to spatial, and temporal changes was solely based on the active community. For community diversity analysis, we used the R package breakaway (v. 4.7.9, [65]) as well as divnet-rs (v. 0.2.1, [66]) to compute total richness estimates (S) as well as Shannon diversity. The advantage over conventional sample-wise calculation methods is that in addition, it computes covariate-wise estimations, as well as variance estimates for each sample. To test diversity estimates between fixed combinations of the covariate-wise differences, we used the function ‘betta’, and ‘betta_random’ of the R package breakaway incorporating the sequencing batch affiliation as a random effect, as well as computing a global *p*-value with an F-test using a bootstrap analysis with 10,000 iterations implemented in the function ‘test_submodel’ in the R package breakaway. Shannon diversity estimates were computed from respective model matrices of co-variates of interest, with 4 replicates, and perturbation set to 0.01, otherwise with default tuning settings. A medium abundant base taxon (groundwater: *Rhodoferax ferrireducens/saidenbachensis*, river: *Limnohabitans* sp.) was used for log ratio computation. Shannon entropy (H′) was computed with the ‘shannon_true’ function implemented in DivNet [67]. Due to the non-linear nature of H′ and inability to compare compositional similarities of a set of species assemblages [68], we additionally calculated the Effective Number of Species (ENS) based on the Shannon entropy as:

ENS(H′) = exp(H′)(1)

with the standard error propagated as:

σ_ENS_ = σ_H′_ × e_H′_ (2)

where σ_ENS_ is the propagated error of the ENS. The effective number of species based on H′ is from here on referred to as Shannon diversity and is defined as the number of equally common species required to produce the same value of H′, or heterogeneity, in a given sample [69]. Pielou’s evenness (J) was calculated as a measure for evenness.

Rare taxa are from here on defined by making up less than 0.01% of the community, occurring in less than 20% of the samples. The core community is characterized by being present in at least 80% of the samples and making up more than 0.01% of the community.

Due to the compositional nature of our data [70], we used the centered-log ratio (clr) transformation on ASV abundances, computed in the microbiome package (v. 1.16.0, [71]), and a non-parametric multivariate analysis of variance (PERMANOVA) using the function ‘adonis2’ implemented in the package ‘vegan’ (v. 2.5-7, [72]) to test for differences in community composition between regions, land use, seasons, correlation of groundwater levels with precipitation (measured as SPI), correlation of groundwater levels with Mur River water levels as SRSI, as well as the probability that the groundwater is influenced by the River Mur. For analysis of species turnover, we excluded samples with fewer than 500 reads. Environmental differences between groups (e.g., land use and region) were calculated as standardized Euclidean distances between samples, whereas differences between groups in community composition were calculated as Aitchison distances. The Aitchison distance is the Euclidean distance between clr-transformed abundances [73], and it is an equivalent to Bray–Curtis, but better suited for compositional data [70,74]. To identify differences in dispersion between groups, we used a permutational analysis of multivariate dispersion (PERMDISP, [75]) using the functions ‘betadisper’, and ‘permutest’ implemented in the vegan package. The vegan function ‘varpart’ was then used to partition the response matrices of Aitchison distances with respect to measured variables [76]. The relevance of parameters was beforehand identified via a distance-based redundancy analysis (db-RDA).

To investigate the relationship between community composition, and environmental parameters we used a Constrained Analysis of Principal Coordinates (CAP) based on the ‘capscale’ function in the R package ‘vegan’ [77]. Prior to CAP, the collinearity of environmental parameters was tested. The scaled environmental parameters were selected by stepwise regression in both directions of variables previously selected via the calculation of the variance inflation factors (VIF) (<10).

To estimate the contribution of various community assembly processes in groundwater and the Mur River, we used the iCAMP package, calculating the beta net relatedness index (βNRI) of the RNA-derived data [24,78]. The contribution of assembly processes is estimated for each bin based on a null model analysis of the taxonomic β-diversities using a modified Raup–Crick metric and the phylogenetic diversity. Bins are here defined by the phylogenetic threshold (ds = 0.2) and a minimum bin size limit (=12). For each bin, the fraction of pairwise comparisons with βNRI < −1.96 is considered as the percentage of homogeneous selection (HoS), those with βNRI > +1.96 as the percentage of heterogeneous selection (HeS), based on the threshold applied previously [79,80]. Next, the taxonomic Raup–Crick diversity metric is used to partition the remaining pairwise comparisons (i.e., |βNRI| ≤ 1.96). The fraction of pairwise comparisons with RC < −0.95 is treated as the percentage of homogenizing dispersal (HD), while those with RC > +0.95 as dispersal limitation (DL). The remaining with |βNRI| ≤ 1.96 and |RC| ≤ 0.95 represent the percentages of drift, diversification, weak selection, and/or weak dispersal designated as ‘drift’ [25]. If selection occurs, we may further differentiate between homogeneous (e.g., caused by similar environmental conditions) and heterogeneous selection (i.e., species performance). In addition, dispersal rates are divided into dispersal limitation (i.e., high compositional turnover caused by a low dispersal rate) and homogenizing dispersal (i.e., low turnover caused by high dispersal rates), leaving all other assembly effects to drift. To test the correlation of the most dominant processes in groundwater and the river to environmental parameters, we applied a Mantel test and a mixed regression model (MRM) as described in Ning et al. [25]. Spatial distances were transformed via a principal coordinates of neighborhood matrix (PCNM) prior to regression analysis, capturing the most variation in latitude and longitude between sites [72]. The relative importance of each process, as well as the environmental variables were either log-transformed or not to explore the best model for each factor. For each measurement, both the mean and variation between sample pairs were used to investigate the correlation with the relative importance of the individual process [25].

To test for differentially relative abundant taxa across different parameters, we used the ‘differentialTest’ function using a Wald test with a false discovery rate (FDR) cut-off of 5%, implemented in the corncob package (v. 0.2.0, [81]). The resulting abundance coefficient estimate is the estimate from the beta-binomial model that models the ASV against a parameter. The Venn diagram of shared taxa across categories of nucleic acid fractions and water types was created with the function ‘ps_venn’ in the MicEco package (v. 0.9.17) that was weighted by relative abundance, computing counts of ASVs as well as percentage of sequences per total sequence number. There has been filtering of the sequences applied beforehand, such as decontamination, filtering of only archaeal and bacterial taxa, and removal of samples with less than 100 reads. We did not apply any further filtering since the tools we use for diversity estimation are dependent on the rare fraction, which is large in groundwater (89% of the ASVs that make up 31% of the reads).

## 3. Results

### 3.1. Physico-Chemical Characteristics of Groundwater in the Mur River Valley

**Figure 2:**
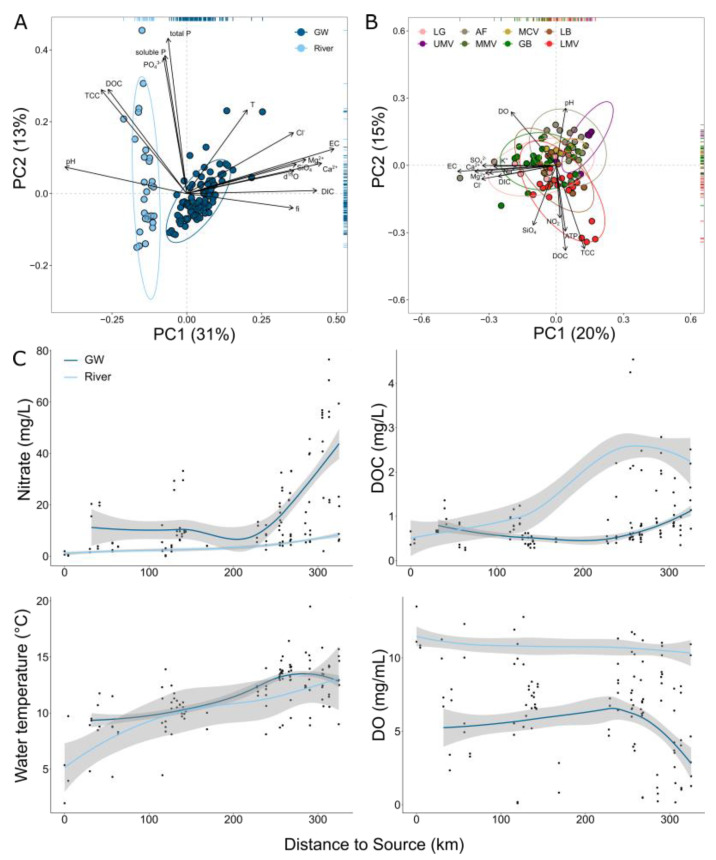
(**A**) Principal component analysis (PCA) based on selected environmental parameters clearly separates groundwater and river water samples. (**B**) Groundwater samples colored according to their origin in terms of sub-regions (groundwater bodies). The ellipses depict the 95% confidence interval ellipses (**A**,**B**). (**C**) Nitrate, DOC, and DO concentrations, as well as water temperature (each sampling site represented by a summer and autumn value), are plotted against the distance to the Mur River source. Grey shading indicates the 95% confidence intervals. (TCC: total cell count; ATP: cellular adenosine triphosphate; DOC: dissolved organic carbon; T: water temperature; DO: dissolved oxygen; EC: electrical conductivity; δ^18^O: stable 1^8^O isotope signature in water; NO_2_: nitrite; PO_4_^3−^: orthophosphate; Na^+^: sodium; K^+^: potassium; Ca^2+^: calcium; Mg^2+^: magnesium; Cl^−^: chloride; SO_4_^2−^: sulfate; soluble P: soluble phosphorus; total P: total phosphorus; SiO_4_: silicate; DIC: dissolved inorganic carbon.)

The physico-chemical characteristics, the concentrations of nutrients, as well as the microbial productivity in terms of prokaryotic cell numbers clearly distinguished groundwater from surface water (Figure 2A). In general, the river has significantly less nitrate, a lower electrical conductivity, and a lower water temperature (*p* adj. < 0.05). On the other hand, the river water contained higher DOC and DO concentrations, and was characterized by a higher pH, and a higher microbial productivity (in terms of cellular ATP and cell count) (Table 1) (*p* adj. < 0.05). Individual features of groundwater bodies are the highest productivity in the Lower Mur Valley (LMV), with high DO concentrations in the Graz Basin (GB) and the Aichfeld (AF). The sub region Aichfeld (AF) delivered groundwater with slightly alkaline pH values (Figure 2B, Table 1). In groundwater, differences between samples from summer and autumn are especially pronounced for temperature, and pH (in autumn, the groundwater has higher water temperatures and a lower pH) (*p* adj. < 0.05), whereas with the river water samples, temperature, pH, and DO are significantly affected by the season (in autumn, the river has lower water temperatures, a higher DO content, and a lower pH) (*p* adj. < 0.05) (Table 1).

In groundwater, the following physico-chemical parameters are changing significantly down the river valley with distance from the Mur River source: There is an increase in groundwater temperature (*p* < 0.05), nitrate concentration (*p* < 0.05), DOC concentration (*p* < 0.05), SiO_4_ concentration (*p* < 0.05), and an increase in the water δ^2^H signature (*p* < 0.05) (Figure 2C). In groundwater, DO is negatively correlated with DOC (r_s_ −0.49, *p* < 0.05) and prokaryotic cell numbers (r_s_ −0.66, *p* < 0.05). Prokaryotic cell numbers are positively correlated with cellular ATP (r_s_ 0.67, *p* < 0.05) and DOC (r_s_ 0.76, *p* < 0.05). On the abiotic side, EC correlates strongly with sodium (r_s_ 0.75, *p* < 0.05), magnesium (r_s_ 0.69, *p* < 0.05), and DIC (r_s_ 0.72, *p* < 0.05) (Figure S2).

In our dataset, ‘agricultural areas’ make up 58% of the land cover, followed by ‘artificial surfaces’ (31%) and ‘forest and semi natural areas’ (11%). Land cover could be connected to some of the environmental parameters, i.e., ‘agricultural areas’ were characterized by high pH, and nitrate concentrations (from fertilization), and ‘artificial surfaces’ (including urban land) showed high EC values (Figure S3). In more detail, nitrate was highest in the Corine land cover categories ‘land principally occupied by agriculture with significant areas of natural vegetation’, ‘broad-leaved forests’, and ‘non-irrigated arable land’, and it was lowest in ‘mixed forests’ and ‘pastures’. DOC was highest in ‘mixed forests’ and ‘broad-leaved forests’ and lowest at groundwater sites located below ‘pastures’ (Table S3).

The influence of surface water to groundwater can be described as follows: groundwater that is likely to receive inflow from the Mur River, as based on groundwater level triangulation, also showed on average a shorter minimum distance to the river than groundwater that is not, weakly, or moderately influenced (Figure S4) (Kruskal–Wallis *p* < 0.05).

### 3.2. Microbial Productivity in Groundwater and River Water

**Figure 3:**
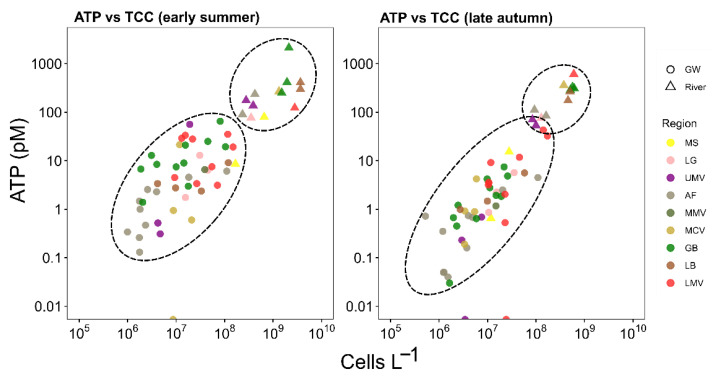
Bivariate plot of the prokaryotic cell counts, and the cellular ATP concentrations of groundwater and river water samples collected in summer (**left**) and autumn (**right**) 2020.

The microbial productivity is estimated from the prokaryotic cell density, and the cellular ATP concentration (cell activity). For both seasons, surface water was found more productive (ATP: median = 175.1 pM (Interquartile range (IQR) = 222.7), cell counts: median = 4.4 × 10^8^ cells L^−1^ (IQR = 6.6 × 10^8^)) than groundwater (ATP: median = 2.5 pM (IQR = 6.6), Cell count: median = 1.05 × 10^7^ cells L^−1^ (IQR = 2 × 10^7^)). The samples from the source of the Mur River (MS) were more similar to surface water productivity in summer and more similar to groundwater in autumn (Figure 3). Overall, the microbial productivity of river water in the upper mountainous reaches, i.e., Lungau (LG) and Upper Mur Valley (UMV), were similar in values to groundwater of the lowermost region of the Mur River transect, the Lower Mur Valley (LMV) (Figure 3). In groundwater, prokaryotic cell counts did not differ between seasons, but cellular ATP concentrations were higher in summer than in autumn (*p* < 0.05). In river water, cell counts were higher in summer (*p* < 0.05), but ATP concentrations are statistically similar in summer and autumn. In autumn, there is a stronger linear relationship (Adj. R^2^ = 0.75) (*p* < 0.05) between ATP and prokaryotic cell density compared to summer (Adj. R^2^ = 0.64) (*p* < 0.05) (Figure 3).

### 3.3. Prokaryotic Diversity and Community Composition in Groundwater and Comparison of the Total and Active Fractions

Prevalence-based contaminant identification was performed independently for each sequencing batch and found 11,326 ASVs and 728 contaminants overall in the dataset. Furthermore, this dataset, composed only of Bacteria and Archaea, consists of ASVs that could be classified to 94% at phylum level, 90% at class level, and 80% at order level. Summary statistics can be found in Tables S4 and S5.

**Figure 4:**
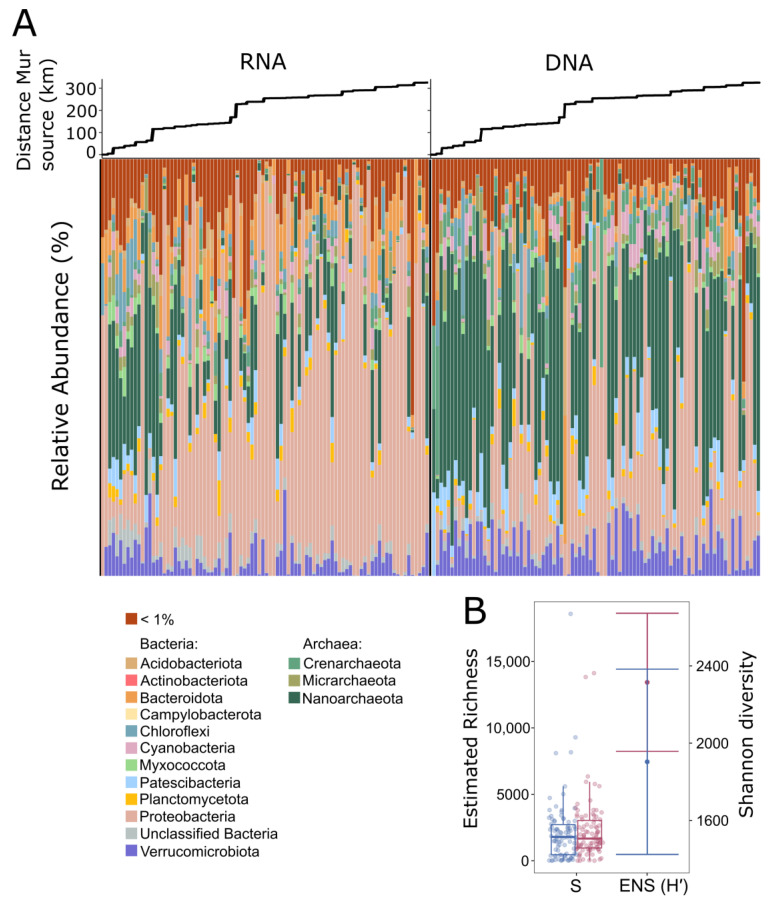
(**A**) Relative abundance plot of total, and active fractions of groundwater prokaryotic communities. Samples were ordered according to their distance to the source of the Mur River, where the source itself is on the far-left side of the plot. (**B**) Total richness estimates (S) are plotted in a boxplot showing differences in RNA (blue)- and DNA (red)-derived communities, as well as covariate-wise mean estimates of DNA- and RNA-derived communities of the Shannon diversity (ENS (H′)) showing error bars as error of the mean.

The taxonomic composition of the prokaryotic communities based on DNA (total community) and RNA (active community) extracted from groundwater in summer and autumn 2020 is visualized in Figure 4A. The majority of sequences are from Bacteria. However, almost half of the bulk community in groundwater is made up of Archaea (42.2% ASVs, 43% of the reads), which become less abundant in the RNA-derived fraction (18% ASVs, 19% of the reads). In both nucleic acid fractions, Archaea became significantly less abundant with increasing distance from the River Mur source (Figure S5).

In groundwater, the most abundant phyla are Nanoarchaeota (DNA: 34.3%, RNA: 14.2%), Proteobacteria (DNA: 30.4%, RNA: 46.4%), Verrucomicrobiota (DNA: 6.2%), and Bacteroidota (RNA: 7.2%), respectively. Of those, Nanoarchaeota and Verrucomicrobiota significantly decrease in the RNA-derived community. Proteobacteria significantly increase with increasing distance from the Mur source in both DNA- and RNA-derived communities (Figure S6). Proteobacteria in groundwater are mainly composed of Gammaproteobacteria (DNA: 83%, RNA: 86% of all Proteobacteria ASVs), which represent 28.8% of the total reads in the DNA fraction and 42.5% in the RNA fraction. Archaeal lineages belonging to unclassified Woesearchaeales, part of the class Nanoarchaeia, are most abundant in the groundwater (DNA: 52%, RNA: 57% of all archaeal ASVs), which represent 24% of the total reads in the DNA fraction and 11% of the total reads in the RNA fraction. Archaeal lineages significantly decreasing over the course of the alpine river transect in the active fraction are unclassified Archaea and representatives of the Deep Sea Euryarchaeotic Group (DSEG), being part of Aenigmarchaeota. Additionally, there is a non-negligible number of unclassified Bacteria found throughout the groundwater communities (DNA: 1.4%, RNA: 2.3% of ASVs), also being significantly more abundant at higher elevations in both the DNA and RNA fraction (*p* < 0.005).

Overall, total estimated prokaryotic richness does not differ significantly between the active and total fraction of the community (RNA, S: 1100 ± 63, DNA, S: 1196 ± 89). The differences in Shannon diversity, however, are significant over all samples, being 18% higher within the DNA fraction of the groundwater (*p* < 0.05) (Figure 4B, Table 2). In short, groundwater species diversity is higher in the DNA fraction, declining in low abundant taxa, with common species starting to play a larger role in the active fraction of the community, as richness does not seem to diminish. In groundwater, 55% of the ASVs and 85.5% of the reads are shared between the active and total fraction (Figure S7).

When testing for overall differences in species turnover between nucleic acid fractions with PERMANOVA (999 permutations), the difference between nucleic acids (DNA and RNA) alone explains around 4.5% of the variation in community composition based on the clr metric.

ASVs showing the largest differences in abundance between nucleic acid fractions over the whole dataset are visualized in Figure S8. All communities show homogeneous dispersion among nucleic acid groups.

### 3.4. Active Prokaryotic Community Diversity between Groundwater and the River

Groundwater and river samples share 22.7% of the ASVs, and 66.7% of the reads (Figure S9), which indicates a high degree of microbial exchange between the two habitats, but also a certain degree of niche specialization. The river only harbors a small fraction of unique ASVs (7.7% ASVs and 4.1% reads), while ASVs unique to groundwater make up 69.6% of the groundwater ASVs, and 29.3% of the reads (Figure S9). The active groundwater core community comprises six taxa making up 16% of the total reads (*Ferribacterium limneticum*, 0.68%; *Actimicrobium* sp., 3.0%; *Rhodoferax ferrireducens*, 2.6% as well as three taxa belonging to *Pseudomonas* sp. (5.8%, 2.5%, 2.2%)), but they are significantly varying in abundance between wells (Kruskal–Wallis; *p* < 0.05). Rare species make up around 89% of the ASVs and 31% of the reads in the groundwater active community. The rare groundwater community comprises taxa belonging to unclassified Bacteria, *Acinetobacter lwoffii*, *Gallionella* sp., unclassified SAR202 clade, unclassified Xanthomonadales, and unclassified Magnetospirillaceae, namely, the five most abundant ones. The river core community comprises thirteen taxa (the five most abundant taxa with percentage of relative abundance in parenthesis are: *Limnohabitans* sp. with 7.9% abundance, followed by *Malikia granosa* (7.8%), *Pseudarcobacter suis* (7.6%), *Rhodoferax ferrireducens* (2.8%), and *Acinetobacter bohemicus* (2.0%)) that represent 28% of the total reads, which vary significantly in abundance between samples (Kruskal–Wallis; *p* = 0.000497). The river ASVs consist of 70.7% rare taxa, which make up 9% of the total sequences.

In the river, the following phyla are significantly more abundant when compared to groundwater: Campylobacterota, Bacteroidota, Fusobacteriota, Armatimonadota, and Cyanobacteria (Figure S10). The observed richness captures around 20% of the total estimated richness of the active fraction in groundwater, and about 35% of total estimated richness in the river samples, respectively (Table S6).

Groundwater (S: 1272 ± 97 ASVs) harbors a significantly higher total estimated richness compared to the river (S: 780 ± 188 ASVs) (*p* = 0.007), as well as a higher Shannon diversity (Groundwater: 1442 ± 11 ENS, River: 339 ± 86 ENS) (*p* < 0.05). DNA-derived communities show a similar pattern. Prokaryotic diversity and complexity are therefore clearly higher in the active community of the groundwater ecosystem compared to the river water.

The active river prokaryotic communities are also significantly different from groundwater in their taxonomic community composition (R^2^ = 0.115, *p* < 0.05), with river communities (57.4 ± 10.5) being on average more similar to each other than groundwater (73.7 ± 11.4) (Wilcoxon: W = 8,406,047, *p* < 0.05) (Figure S11). Using a distance-based redundancy analysis, we identified potential environmental drivers (out of physico-chemical variables, and nutrients) that cause the differences between surface water and groundwater communities. Taxonomic differences are largely driven by variation in DO, pH, SiO_4_, water temperature, DIC, DOC, δ^2^H, sodium, and potassium which explains around 17% (Adj. R^2^) of the variation in taxonomic community composition between groundwater and river water.

#### 3.4.1. Community Assembly Processes Differ between the River and Groundwater

To determine which assembly processes shape the prokaryotic communities in groundwater and the Mur River, prokaryotic 16S RNA sequences were grouped into a total of 110 phylogenetic bins. Pairwise comparison of all river sampling sites (within-group, number of comparisons = 1265), as well as between groundwater sites (*n* = 13,505) were evaluated. Our results show large differences regarding the assembly processes of prokaryotic river water communities in comparison to groundwater. Deterministic homogeneous selection (HoS, similar habitats harbor similar communities), dispersal limitation (DL, high compositional turnover between sites, mainly caused by low dispersal rates) and ecological drift (DR, stochastic changes in relative abundance) are the most relevant processes for structuring the communities. In general, the impact of homogeneous selection (HoS) is significantly greater (Wilcoxon: W = 539,030, *p* < 0.05) in the river than in groundwater. In contrast, dispersal limitation (DL) plays a greater role for the groundwater prokaryotes than for the lotic communities (Table 3).

#### 3.4.2. Environmental Factors Influence Community Assembly Processes in Groundwater

**Figure 5:**
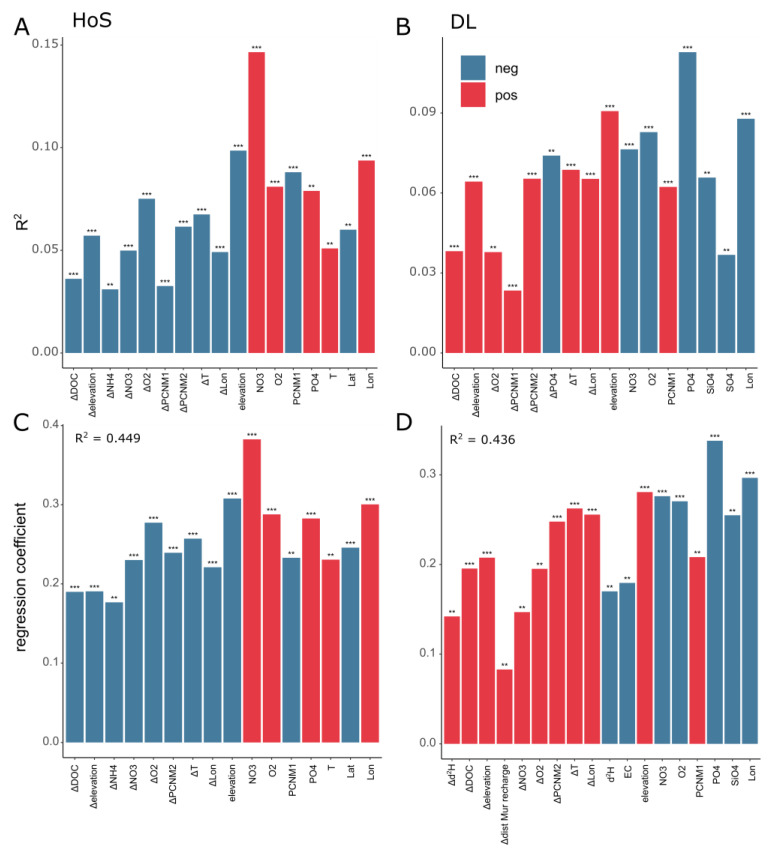
Correlations of environmental variables showing factors with significant correlations for HoS (**A**) and DL (**B**) based on a Mantel analysis. Multiple regression on distance matrix (MRM) for HoS (**C**) and DL (**D**) with regression coefficient on the *y*-axis and R^2^ shown based on the model from the Mantel test, which was based on log-transformed data. Negative correlations are colored in blue, and positive are colored in red. Correlation was based on the difference (Δ) or the mean of the variables between each pair of samples (*** *p* < 0.01; ** *p* < 0.05).

Environmental factors that correlate with the most important assembly processes in groundwater were tested via a Mantel test and a mixed regression model (MRM) under both seasons [25]. These two processes were dispersal limitation (DL) and homogeneous selection (HoS) (Table 3). Both processes could be linked to environmental factors related to nutrient concentrations, as well as geographical distance, which could explain around 44% of the variation of both HoS and DL (Figure 5). In contrast, environmental factors were able to explain much more of the variation (R^2^ > 0.8) in community assembly processes in each HoS and DL in the river (Figure S12). In groundwater, HoS correlated most strongly with nitrate concentrations and elevation (R^2^ > 0.098) and more weakly with longitude, the first two principal coordinates of neighborhood (PCNM) as well as water temperature and dissolved oxygen concentrations (R^2^ > 0.06). DL on the other hand correlated most strongly with phosphate concentrations, as well as elevation (R^2^ > 0.09), and more weakly with longitude, dissolved oxygen, as well as nitrate concentrations and between-sample differences in phosphate concentrations and water temperature (R^2^ > 0.068). Even though these correlations are significant, they are quite weak (R^2^ < 0.1).

The most important contributions of different variables were determined with an MRM. Regarding processes of HoS, again, nitrate concentrations, as well as elevation, had the largest effects (Figure 5C). DL had an opposite relationship to various environmental factors (e.g., nitrate, phosphate, longitude, elevation, and dissolved oxygen), out of which phosphate and longitude had the largest effects on DL (Figure 5D). In summary, DL is positively related to elevation (coinciding with the geographic location of the groundwater sites along the Mur Valley). This means that within alpine groundwater (in this dataset at high elevation, high latitude, and low longitude), DL plays a more important role in community assembly, whereas in the alpine foreland (here at lower elevations, lower latitude, and higher longitude), HoS is the prevalent process for community assembly that is mostly related to changes in nutrient concentrations.

### 3.5. Ecological Concepts

**Figure 6:**
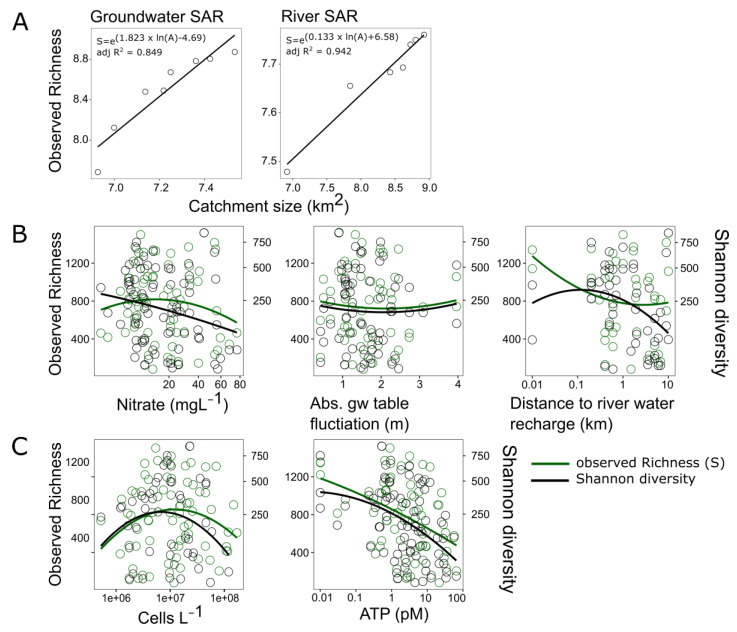
Bivariate SAR linearized log–log power function plot of cumulatively added groundwater and Mur River ASVs (RNA fraction) with increasing catchment area. Groundwater catchment area and observed richness were cumulatively summarized from the groundwater regions closest to the source of the River Mur (LG) to the groundwater regions furthest away (LMV), only adding taxa that were not found in the previous groundwater region. The Mur River catchment sections (from top to bottom: MUR SBG, MUR I, MUR II, MUR III, MUR VI, Laßnitz, MUR V) were also summarized from the source of the Mur River in the Hohe Tauern to the area furthest downstream (MUR V) (**A**). Intermediate disturbance hypothesis (**B**) and productivity–diversity concept (**C**): Biplot of square root-transformed Shannon diversity (black), and observed richness (S; green), as well as square root transformed nitrate (mg L^−1^), log10 transformed Mur inflow distance (km), and untransformed groundwater level fluctuation between absolute max and min since the year 2000 (m), fitting a 2nd-degree quadratic regression line of the groundwater data, as well as log10 transformed prokaryotic cell counts (cells L^−1^) and cellular ATP (pM) to meet criteria of a normal distribution.

The species area relationship (SAR) applied for groundwater and river water prokaryotes follows the common assumption that the number of species is a positive function of the area sampled (Figure 6).

The way groundwater diversity (observed richness and Shannon diversity) is linked to external disturbances and impacts from the surface is visualized in the context of the ‘intermediate disturbance hypothesis’ (Figure 6). Out of the relationships tested, observed richness does not show a statistically significant relation to any of these disturbances. However, the Shannon diversity revealed a negative linear relationship with nitrate concentrations (adj. R^2^ = 0.04) (Figure 6B).

The relationship between observed richness and cell numbers is significantly explained by a quadratic model (adj. R^2^ = 0.022) and is linearly negatively related to cellular ATP (adj. R^2^ = 0.15). The Shannon diversity does not vary with cell numbers but shares a significant linear negative relationship with cellular ATP (Adj. R^2^ = 0.17).

This means that in our groundwater dataset, diversity is principally highest at lower levels of disturbance. Investigating the productivity–diversity concept regarding our data, the Shannon diversity is not changing with cell numbers, while observed richness is highest at intermediate levels of cell density. Observed richness and the Shannon diversity are changing with the concentration of intracellular ATP, both being lowest when ATP is highest.

### 3.6. Temporal Shifts in Diversity and Community Dynamics in the Active Groundwater Fraction

Comparing total estimated richness between summer and autumn, groundwater has significantly higher richness in autumn (S: 1626 ± 121) compared to summer (S: 1062 ± 164) (*p* < 0.05). Moreover, the Shannon diversity in groundwater is higher in autumn as well (summer: 622 ± 96 ENS, autumn: 1847 ± 67 ENS) (*p* < 0.05), which hints at a more evenly distributed community towards autumn, which is richer in species. In the DNA fraction, the picture is similar, with no difference in the richness between seasons but higher diversities in autumn (Figure S13).

### 3.7. Regionality and Land Use Influence Diversity in the Active Groundwater Community

**Figure 7:**
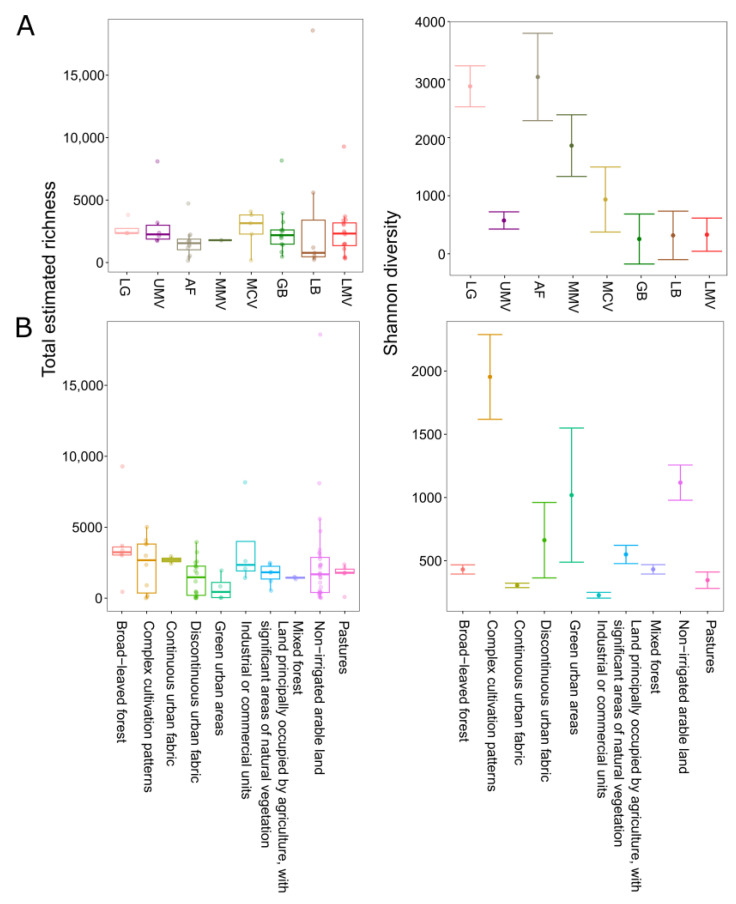
(**A**) Diversity metrics of groundwater prokaryotic communities between regions and (**B**) related to different land use types. Total estimated richness is depicted in form of boxplots showing median values as midline, first, and third quantiles as edges, and whiskers representing the max/min values. Shannon diversities are shown as effective species numbers with error bars showing the mean estimates from covariate-wise estimations ± SE.

Specifically, in groundwater, there are overall regional differences in total estimated richness (*p* = 0.04), where richness is highest in the region Mur Cross Valley (MCV) (S: 2175 ± 461) and lowest in the Leibnitz Basin (LB) (S: 500 ± 398). There are also overall differences in the Shannon diversity between regions (*p* < 0.05), which becomes gradually less towards lower elevations, being highest in the region Aichfeld (AF) (3072 ± 85 ENS), and lowest in the region Graz Basin (GB) (259 ± 46 ENS) (Figure 7A). In principle, groundwater located in the alpine regions shows a higher species diversity and complexity, with exception of the Upper Mur Valley (UMV) with only a few samples collected, than regions in the alpine foothills, where there are a few abundant species dominating the community. UMV is probably less diverse, as the communities are not as evenly distributed compared to the region Lungau (LG) (*p* < 0.05) and is more similar in evenness to wells located in the downstream part of the Mur Valley transect of Graz Basin (GB), Leibnitz Basin (LB), and the Lower Mur Valley (LMV). A similar trend can be observed in the DNA fraction (Figure S14).

In groundwater, total estimated richness is comparable over all land use types, but the Shannon diversity is significantly different between categories of land use (*p* < 0.05). The groundwater Shannon diversity is highest in wells located beneath ‘Complex cultivation patterns’ (2503 ± 163 ENS), and lowest in wells beneath ‘Industrial or commercial units’ (164 ± 35 ENS) (Figure 7B). Results are similar for the richness and Shannon diversity in the DNA-derived community (Figure S15).

### 3.8. Land Use Affiliation, and Regionality as a Major Factor for Species Turnover

In groundwater, the categories land use and regionality explain most of the variation in the taxonomic community composition of the prokaryotic communities. The PERMDISP analysis, designed to test if some groups are more variable than others, reveals significant differences for season, land use, and groundwater level correlation with 9–12-month SPI (Table 4). Notably, the heterogeneity of overall environmental parameters between seasons does not seem to cause the observed patterns of beta diversity. Differences in environmental conditions, however, could offer an explanation for the multivariate homogeneity of group dispersions between land use categories (*p* < 0.05), and strengths of well level correlation with 9–12-month average precipitation (*p* < 0.05).

**Figure 8:**
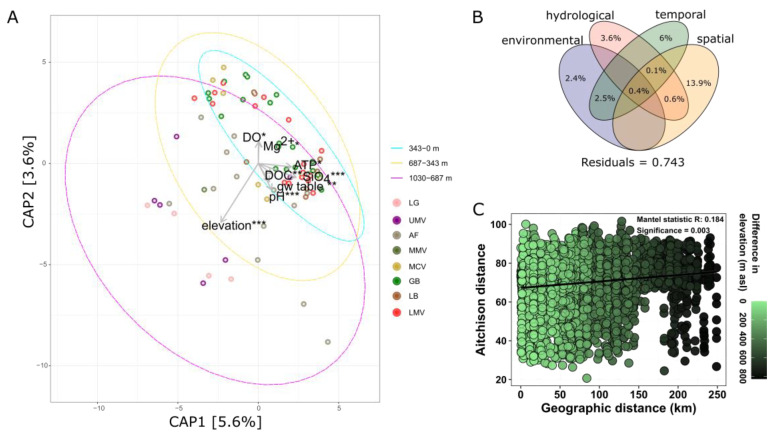
(**A**) Ordination of species turnover with a constrained analysis of principal coordinates (CAP) based on clr-transformed abundances between groundwater wells in association with respective environmental parameters (*** *p* < 0.001, ** *p* < 0.01, * *p* < 0.05). (**B**) Venn diagram showing the proportions of variation explained by four explanatory factors: environmental factors, hydrological factors, temporal, and spatial factors. (**C**) Biplot of the Aitchison distance and geographic distance depicting the distance decay relationship with circles colored according to the difference in elevation between sample pairs, showing Spearman correlation coefficient (R) as well as *p* value.

Next, we plotted key physico-chemical parameters and nutrients using a con-strained analysis of principal coordinates (CAP) of the Aitchison distances. The taxonomic composition is significantly explained by differences in elevation, cellular ATP, pH, SiO_4_, groundwater level, DO, Mg^2+^, and DOC (Adj. R^2^ = 0.108), where elevation (Adj. R^2^ = 0.037) and pH (Adj. R^2^ = 0.036) alone explain the most variation in taxonomic composition of groundwater prokaryotic communities (Figure 8).

Based on a variation partitioning analysis, excluding variables that are correlating beforehand, differences in significant environmental factors (pH, K^+^, DO) explain 2.4%, while the significant hydrological fraction (SRSI, and probability that groundwater is influenced by the river water) explains 3.6%, the temporal factor season explains 6%, and spatial factors (region, land use) explain 13.9% of the variation in prokaryotic community composition. Environmental, hydrological, temporal, and spatial factors are still significant when controlling for the respective others. The dataset covers a distance gradient of almost 300 km. The distance–decay relationship (Figure 8) depicts an overall positive correlation that indicates a higher species turnover between samples as the distance increases between them (Figure 8). These results suggest that spatial components such as region, land use, geographic distance, and elevation account for most of the variability in community turnover. Furthermore, communities originating from the low altitude section (0–343 m a.s.l.) seem to be most similar to each other, compared to communities derived from the alpine region (Figure 8). This indicates a higher structural heterogeneity in alpine aquifers and some separation of higher altitude groundwater bodies (>400 m a.s.l.), where members of the archaeal phyla Nanoarchaeota, Aenigmarchaeota, Micrarchaeota, as well as unclassified Archaea, together with bacterial phyla WOR-1, Patescibacteria, Chloroflexi, Verrucomicrobiota and unclassified Bacteria are significantly more abundant, from groundwater bodies in the lowland (200–400 m a.s.l.) Proteobacteria gain significantly in abundance (*p* < 0.05).

## 4. Discussion

In our study, we collected and characterized shallow groundwater and corresponding river water from eight regions along a 300 km river valley stretch with a focus on at the active microbial diversity and community. The dataset was further explored in relation to hydrochemical composition and physicochemical conditions, microbial productivity, as well as community composition with respect to aspects of altitude, season, and land use, as well as local and regional hydrology influenced by precipitation and interaction with the river. We investigated how the groundwater prokaryotic communities differed in their active (RNA derived) and total (DNA derived) composition in terms of structure, richness, and diversity. The active batch of the prokaryotic communities was then analyzed regarding different community assembly processes. Moreover, ecological concepts established in macroecology and partly in microbial ecology of soil ecosystems and surface waters were tested for the Mur Valley groundwater ecosystem.

Groundwater is often perceived as an environment stable in hydrological and physico-chemical conditions carrying a rather low productivity and biodiversity [2]. Due to protective overlying soil layers and extended water residence times, groundwater systems are usually more self-contained and less affected by external seasonal disturbances (e.g., extreme weather events), land use, and accidental pollution when compared to surface ecosystems such as rivers and lakes. In contrast, several recent studies provide solid evidence that aquifers in karst and shallow unconsolidated alluvial and glacio-fluvial sediments exhibit quite some dynamics in environmental conditions translating into dynamics in microbial community characteristics, including community composition and productivity [13,35,82,83]. Evidence is also growing that although depleted in readily biodegradable organic carbon and poor in energy, groundwater ecosystems may harbor highly diverse microbial communities absolutely comparable to surface waters in terms of richness and diversity [3,84,85].

A river corridor originating from the mountainous region extending into lowlands offers the possibility to systematically study microbial community composition along various gradients, i.e., a change in elevation and temperature regime, variation in the concentration and quality of natural organic matter, an increase in land use types and intensity translating into an increase in nutrients and pollutants, and a change in microbial diversity and productivity in surface waters and groundwater [86,87,88,89]. Indeed, groundwater downgradient of the source of the River Mur at about 2000 m a.s.l. continuously increased in temperature, DOC, and nitrate concentrations (Figure 2). In addition, silicate is enriched in groundwater and, to a lesser degree, in the Mur River and tributaries over the course of the valley, with the Laßnitz River especially being a contributor draining a silicate-rich area. The strong relationship between DOC and DO concentrations suggest a high bioavailability of DOC [90] in the Mur Valley groundwater. Harjung et al. (2023) [89] pointed at a correlation between groundwater DOC quality and concentration with prokaryotic biomass in the large river valleys of Austria, including the Mur River valley.

As a proxy for microbial productivity, we used prokaryotic cell density and cellular ATP. In general, groundwater and surface water differed significantly in productivity, with groundwater being less productive (Figure 3). Within groundwater, productivity increased from the alpine region to the lowlands, and was lower in greater depth (e.g., in the Aichfeld and Graz Basin region) compared to shallow groundwater (e.g., in the Lower Mur Valley). Prokaryotic cell densities observed in groundwater exhibited values typical for groundwater ranging from 10^6^ to 10^8^ cells L^−1^ (Figure 3) and only in exceptional cases hint at serious pollution [2,46]. Temporal differences in productivity were evident between groundwater collected in summer and autumn. This variation can be related to seasonal inputs of energy from the surface (i.e., winter and spring are the periods of main groundwater recharge), a change in microbial community composition, and/or a change in physiological state [47]. Evidence for the latter comes from elevated cellular ATP values in summer when compared to autumn, with prokaryotic cell densities showing no significant differences between seasons (Figure 3). In case of an increased availability of organic matter in parallel to a lack of an essential nutrient such as phosphorus, cells may increase physiological activity (and thus ATP content) without cell reproduction [91].

Based on molecular analysis, differences between the active (based on total RNA) and the total fraction (based on total DNA) of the prokaryotic communities in groundwater are especially pronounced in the different relative abundance of Bacteria and Archaea (Figure S8). Since the total and active fraction are equally rich in species (ASVs), but the Shannon diversity is higher in the total fraction, the difference in abundance of some prokaryotic groups may explain this outcome. This hints at both communities, the total and the active, being differently structured. In groundwater, Archaea make up >40% of the species in the total, DNA-derived fraction, whereas in the active community, Archaea account for <20%. This indicates that a large proportion of Archaea in the groundwater obviously is inactive, dead or in a dormant state. Overall, the most abundant archaeal lineages in groundwater are members of unclassified Woesearchaeales. Members of Woesearchaeales (Candidatus Woesearchaeota) have been discovered from a diverse variety of aquatic habitats [92,93,94] and are hypothesized to have a symbiont and/or fermentation-based lifestyle. Their presence in the active fraction of the groundwater microbiome suggests that they likely take part in organic carbon cycling; however, a detailed process understanding is lacking. In the RNA-derived fraction, there is an increasing presence of Proteobacteria, mainly composed of Gammaproteobacteria, comprising almost half of the reads of the active groundwater community. No matter the nucleic acid fraction, Proteobacteria comprise the most abundant phylum present, as has also been observed in other groundwater studies [95,96,97]. A more extensive picture into the groundwater microbiome could emerge if microbial communities attached to the aquifer matrices as well as microeukaryotes were also included in a future analysis, since they will contribute significantly to compositional aspects as well as functions [98].

Groundwaters and surface waters analyzed in this study were fundamentally different in their species diversity in both the active and total fraction, with groundwater being on average 76% more diverse and 38% richer in prokaryotic taxa. A comparable or even higher microbial diversity in groundwater and surface waters, respectively, have been found repeatedly in modern studies [85], mainly attributed to the improved sensitivity of molecular tools for rare taxa. The active river prokaryotic biome shares 67% of the reads and 23% of the ASVs with the active groundwater prokaryotic community, with groundwater harboring a higher fraction of uniquely occurring taxa. The active groundwater prokaryotic community comprises six core taxa, predominantly consisting of Pseudomonas species. The larger proportion of the active groundwater community is composed of species with a relative low abundance and composed of many unclassified Bacteria, suggesting the presence of yet undescribed bacterial lineages in the groundwater. These rare microbial taxa in groundwater shall be addressed in more detail in future studies. There is evidence that taxonomic minority members in microbial communities may be responsible for major activities [42,99]. The active core community in the river consists of 13 species and show a relatively lower proportion of rare taxa compared to the total fraction. This confirms the assumption that species that are abundant within one site tend to occupy many sites, while those that are locally rare will not be detected in many sites [100]. Physico-chemical drivers of the variation in groundwater and river prokaryotic community composition are DO, pH, SiO_4_, (R^2^ > 0.2), followed by water temperature, concentrations of DIC and DOC, δ^2^H (water origin), and Na^2+^ and K^+^ concentrations (Figure S11), leaving around 83% of the variation in community composition unexplained. Other studies investigating the influence of environmental parameters on microbial community composition also found low degrees of explanation [22]. This unexplained proportion of variation can be related to other, yet unmeasured environmental factors, the huge spatial heterogeneity with a low spatio-temporal resolution in sampling, dispersal dynamics (e.g., community assembly linked to stochastic processes), and/or methodological shortfalls [80].

We show that the assembly of prokaryotic communities was shaped by a combination of environmental selection of similar local conditions, dispersal limitation (DL), and ecological drift. Considerable differences in community assembly processes dominating in groundwater and river water habitats were found (Table 4). Previous studies investigating prokaryotic communities in the subsurface [83,101] provide further evidence for homogeneous selection (HoS) and DL to be dominating community assembly processes.

DL was most important in groundwater, meaning that in the absence of selection, prokaryotic communities have fewer taxa than expected by chance. A low rate of dispersal of taxa can happen when habitats or sites are spatially not connected, for instance, in cases of limited groundwater flow between aquifers [102]. DL does play a role for the river prokaryotic assembly as well, for instance, by limiting the turnover of species along the flow path of the river in the downstream direction. However, species assembly processes in the river are to a larger extent influenced by HoS, likely caused by homogeneous biotic and abiotic environmental conditions, leading to a more similar community structure than expected by chance. In accordance with Danczak et al. [101], we found that the groundwater, which experiences higher turnover of taxa than the river, is more strongly affected by stochastic processes, such as DL. On the other hand, little variation in community compositional turnover between river sites can dominate, if communities occur in the same selective environment and if selection is strong.

The longitudinal change in assembly processes (HoS and DL) reflects the hydrogeomorphic conditions along the Mur River valley. This could partly be related to the land management, as reoccurring and predictable environmental conditions have been shown to cause homogeneous selection [103]. It seems that homogeneous selection (HoS) is a more important community assembly process in the alpine lowland, where it is mostly driven by changes in nutrient concentrations (phosphate, nitrate) and oxygen availability (Figure 5). In the alpine region, however, prokaryotic communities are more strongly influenced by dispersal limitation (DL), eventually caused by existing physical barriers (e.g., mountains, narrow valleys) and/or possibly altered hydrological conditions affecting the groundwater flow rates.

Making use of our unique dataset, we tested a selection of ecological concepts that are well established in macroecology but controversially debated and hardly validated in microbial ecology of soil ecosystems and surface waters [35], i.e., the species–area concept (SAR), which describes the relationship between species richness as a function of habitat size [36], the intermediate disturbance hypothesis, which assumes microbial phylogenetic and functional diversity peaking at intermediate intensities or frequencies of disturbances [38,104,105], and finally, the productivity–diversity concept, which assumes an increase in species richness and diversity with increasing available energy, though similarly to disturbances, a decline in richness and diversity happen in systems which are over-stressed and eutrophicated [40]. None of the concepts have been challenged with a solid groundwater dataset [2,35]. First, the species–area relationship holds true for groundwater and river water. A cumulative increase in prokaryotic richness was observed with increasing catchment size (Figure 6). The intermediate disturbance hypothesis did not prove similarly valid for the conditions tested. As disturbances, we tested (i) nitrate as a proxy for agricultural land use and the input of fertilizers and pesticides, (ii) the absolute groundwater level fluctuations as proxy for the hydrological dynamics aquifer vulnerability, and (iii) the distance to the river as a proxy for surface water impact to groundwater. These disturbances (in the range observed) did not reveal a significant quadratic relationship with prokaryotic richness in groundwater. Only the Shannon diversity was negatively related to the intensity of agricultural land use, as classified by nitrate concentration. The intermediate disturbance hypothesis has been frequently questioned and refuted in other studies and might not hold true for every type of ecosystem and all kinds of communities [106]. On the other hand, the influence of available energy and resulting productivity as assessed by total prokaryotic cell counts showed a quadratic relationship with prokaryotic richness, being highest at an intermediate prokaryotic cells density (~10^7^ cells L^−1^). Moreover, we observed linear relationships with both the prokaryotic richness and Shannon diversity decreasing proportionally with increasing ATP concentrations (Figure 6). This could be related in part to the fact that pristine aquifers in the alpine region have high habitat heterogeneity and low productivity, which together lead to higher species diversity. In conclusion, there is proof for the SAR, there is support for a productivity–diversity relationship, with highest richness and diversity at lowest energy levels, and intense agricultural activities represented by high nitrate concentrations (but putatively also by elevated pesticide concentrations) had a negative effect to prokaryotic diversity (Figure 6).

Similarly to a few other studies [82], our work revealed temporal dynamics in groundwater prokaryotic diversity, being higher in autumn where communities were found more evenly distributed and richer in taxa.

In addition to temporal dynamics, there are significant spatial changes in prokaryotic community composition. Along the alpine to lowland river corridor, prokaryotic diversity is altered, being overall higher at higher elevations (Figure 7). In alpine aquifers, environmental connectivity increases downgradient, and groundwater bodies at lower altitudes experience unification and anthropogenic degradation with multiple pressures acting in a concerted way, obviously negatively affecting microbial diversity. The alpine region harbored a more diverse and complex prokaryotic community in its groundwater, hinting at the possibility that mountainous areas are not only important biodiversity hotspots for other domains of life [12,107], but also for prokaryotes living in the aquatic realm below ground. In alpine groundwater, Archaean lineages especially seem to play a yet unexplored role with unclassified Archaea, as well as members of the Aenigmarchaeota (DSEG) being highly abundant. Aenigmarchaeota are one of the earliest diverging lineages within the DPANN superphylum meanwhile discovered in a wide range of habitats, and often found to have a symbiotic lifestyle [108].

We looked at land use information as another important spatial and temporal driver that not only affects the surface environments and underlying soils but may significantly shape groundwater conditions and communities [8,109,110]. Groundwater located beneath areas classified as ‘complex cultivation patterns’ and ‘pastures’ exhibited the highest species diversity, being described as a mosaic of small plots of land consisting of different types of cultivation with occasional houses and gardens, and pastures combining all those areas where grassland and pasture farming is practiced. Lowest diversity on the other hand was discovered from groundwater located beneath ‘Industrial or commercial units’ (e.g., factories, warehouses, and storage facilities typically located in urban or suburban areas). Besides that, ‘forest and semi natural areas’ as part of the broader Corine land cover category, harbored significantly higher abundances of some archaeal lineages, as well as the bacterial phylum Dependentiae, which contains as yet mostly uncultured Bacteria found from a wide variety of environments, mainly discovered from metagenomic studies [111]. Differences in the prokaryotic species turnover (beta diversity) between groundwater habitats is mainly driven by the different land use types, but also by the characteristics of the different groundwater bodies, i.e., the regions. Considering the elevation gradient that is represented by the various regions, groundwater bodies at lower elevations (200–343 m a.s.l.) are more similar to each other than groundwater bodies found at higher elevations (Figure 8), where dispersal of prokaryotic taxa is more limited (Figure 5). Land use and region (as a summary of hydrogeological and climatic regional characteristics) explain by far most of the variability in taxonomic prokaryotic community composition in groundwater, while all physicochemical and microbial variables tested in this study explain only a much smaller fraction of variation.

## 5. Conclusions

Microbial diversity and controlling assembly mechanisms in aquatic systems are an active area of research. However, the groundwater ecosystem has often been overlooked, although its understanding is crucial due to its potential impact on human health and ecosystem functioning, as well as its important role in the overall water cycle. Yet, high-throughput gene amplicon sequencing of both the metabolic potentially active (RNA-derived) and total (DNA-derived) groundwater microbial communities, their comparison, and diversity analyses based on the metabolically active fraction have been few.

In this study, the active groundwater community was found to be largely composed of Proteobacteria (mainly Gammaproteobacteria), whereas the archaeal fraction was dominated by unclassified Woesearchaeales. This also indicates that members of Archaea take an active part in the energy turnover of groundwater environments. Moreover, the groundwater harbors relatively more rare taxa than the river, which is composed of many unclassified Bacteria, suggesting the presence of bacterial lineages with unknown functions that are yet to be described. Several studies have highlighted the importance of unclassified Prokaryotes including the bacterial CPR group and the archaeal DPANN taxa. These microorganisms seem to be abundant in the subsurface and are characterized by a minute cell size and reduced genomic repertoire. Metagenomic and metatranscriptomic analysis have shown a coupling of processes (i.e., metabolic hand-off) and the importance of autotrophic lifeforms in the subsurface. Still, metabarcoding remains the method of choice for rapid and representative microbial community analysis.

The complexity of groundwater habitats in terms of physico-chemical, hydrological, and geological heterogeneities in combination with the limitations of groundwater sampling requires careful analysis and interpretation. In general, aggregate parameters such as land-use and geomorphic regions were more reliable in explaining variation of the community composition, than variation of individual parameters. Current community assembly models gave further evidence that dispersal limitation and homogeneous selection are the driving processes in groundwater. Here, dispersal limitation seems to play a larger role at higher altitudes, whereas homogeneous selection explains a larger share in the alpine foothills, where it is related to the availability of nutrients. The alpine region is also more diverse and richer in prokaryotic taxa, with some unclassified Archaea and early diverging archaeal lineages being highly abundant, again underlining the great need to study and appropriately protect this unique habitat, including its groundwater reservoirs.

## Figures and Tables

**Table 1:** Key physicochemical features of groundwater (GW) and river water samples collected in the River Mur Valley in summer and autumn 2020. Data are means ± SD.

	Regions	N	Altitude (m a.s.l.)	T (°C)	pH	DO (mg/L)	EC (µS/cm)	NO_3_ (mg/L)	DOC (mg/L)
GW	LG	6	1032 ± 13	9.8 ± 1.2	6.8 ± 0.4	5.8 ± 2.2	785 ± 124	13.9 ± 7.9	0.94 ± 0.27
	UMV	6	834 ± 89	9.2 ± 1.5	7.2 ± 0.2	4.6 ± 0.9	377 ± 155	5.9 ± 3.0	0.39 ± 0.15
	AF	20	653 ± 65	10.3 ± 0.7	7.1 ± 0.4	6.2 ± 2.3	515 ± 267	11.6 ± 9.6	0.54 ± 0.22
	MMV	2	584 ± 0	9.3 ± 0.8	7.7 ± 0.1	1.8 ± 1.2	413 ± 14	4.0 ± 0.0	0.46 ± 0.05
	MCV	8	402 ± 19	11.9 ± 0.6	7.3 ± 0.2	6.8 ± 1.3	599 ± 102	9.1 ± 2.9	0.54 ± 0.16
	GB	22	350 ± 21	13.7 ± 1.0	7.2 ± 0.3	6.1 ± 2.2	700 ± 154	17.8 ± 9.1	0.60 ± 0.12
	LB	8	280 ± 6	13.3 ± 1.3	7.2 ± 0.2	4.7 ± 3.8	660 ± 112	23.8 ± 15.2	0.84 ± 0.32
	LMV	18	256 ± 26	13.0 ± 1.5	6.8 ± 0.4	3.7 ± 2.4	546 ± 189	41.1 ± 21.7	1.01 ± 0.39
	Autumn	45	-	12.5 ± 2.1	7 ± 0.4	5.2 ± 2.5	602 ± 219	20.6 ± 16.9	0.66 ± 0.35
	Summer	45	-	11.4 ± 1.6	7.2 ± 0.4	5.5 ± 2.6	585 ± 201	18.4 ± 17.5	0.72 ± 0.29
	Agricultural areas	52	516 ± 247	11.6 ± 2	7.1 ± 0.3	5.8 ± 2.3	562 ± 161	20.9 ± 19.9	0.65 ± 0.29
	Artificial areas	28	454 ± 208	12.6 ± 1.7	7.1 ± 0.4	6.1 ± 2.1	680 ± 271	17.5 ± 11.5	0.63 ± 0.29
	Forest and semi natural areas	10	364 ± 217	12 ± 2	6.9 ± 0.5	1.3 ± 1.1	515 ± 162	18 ± 15.6	1.08 ± 0.31
River									
	Autumn	14	-	7 ± 2.5	7.8 ± 0.1	11.4 ± 0.6	223 ± 69	4.4 ± 2.6	1.5 ± 0.7
	Summer	14	-	12.7 ± 3.3	8.1 ± 0.2	10.2 ± 1	196 ± 76	3.3 ± 2.2	1.8 ± 1.3

**Table 2:** Groundwater alpha diversity metrics of estimated total richness (S), Shannon index (H′), effective number of species based on the Shannon index ENS (H′), and Pielou’s evenness (J). Significant differences are highlighted in bold. Values are means ± SE.

	Total Estimated Richness (S)	Shannon Index (H′)	ENS (H′)	Evenness (J)
RNA	1261 ± 81	**7.55 ± 0.009**	**1903 ± 30**	**0.98 ± 0.002**
DNA	1379 ± 114	**7.75 ± 0.012**	**2314 ± 38**	**1.00 ± 0.004**

**Table 3:** Proportions as percentages of community assembly processes in groundwater and the river.

	Heterogeneous Selection (HeS)	Homogeneous Selection (HoS)	Dispersal Limitation (DL)	Homogenizing Dispersal (HD)	Drift (DR)
River	0.41%	55.80%	17.54%	1.75%	24.98%
GW	3.09%	34.16%	37.86%	3.17%	22.36%

**Table 4:** PERMANOVA and PERMDISP analysis of the effect of sampling region, season, land use, correlation of groundwater levels with precipitation, and Mur River water levels on beta diversity of prokaryotic communities measured by clr (999 permutations) (SRSI: Standardized River Stages Index, SPI: Standardized Precipitation Index).

PERMANOVA				PERMDISP	
Taxonomic (CLR)	R^2^	F	*p*-Value	F	*p*-Value
Land use	0.178	1.998871	0.001	2.0506	0.044
Region	0.139	2.006402	0.001		
Season	0.073	7.343749	0.001	8.5672	0.007
SRSI	0.047	1.594799	0.001		
SPI 9–12 months	0.044	1.469684	0.001	5.4924	0.001
Recharge probability Mur	0.038	1.294373	0.012		

## Data Availability

Gene amplicon sequencing data, generated by the JMF under Project IDs JMF-2009-1 and JMF-2106-09, were deposited under the BioProject accession number PRJNA926931.

## Supplementary Materials

The following supporting information can be downloaded at: https://www.mdpi.com/article/10.3390/microorganisms11030779/s1.
