# Supplementary material for: From the Mountain to the Valley: Drivers of Groundwater Prokaryotic Communities along an Alpine River Corridor
Source: Microorganisms. 2023 Mar 17;11(3):779. doi: 10.3390/microorganisms11030779 (PMC10055094; doi:10.3390/microorganisms11030779)
Supplement: Supplementary file 1 [file microorganisms-11-00779-s001.zip › microorganisms-2213304-supplementary.pdf]

Table S1. Coordinates of sampling sites along the Mur River valley (GW = Groundwater).

sample	lat	lon	Type
3	46.71855	15.90023	GW
4	46.73456	15.90251	GW
5	46.70189	15.75395	GW
6	46.69929	15.75902	GW
7	46.71136	15.7522	GW
8	46.70709	15.65988	GW
9	46.71236	15.6547	GW
10	46.72337	15.65283	GW
11	46.73486	15.67377	GW
15	46.80074	15.54768	GW
19	47.07745	15.44345	GW
21	47.07269	15.44565	GW
22	47.04605	15.43916	GW
24	47.05	15.42218	GW
26	47.07607	15.41315	GW
27	47.06034	15.432	GW
28	46.96718	15.48592	GW
31	46.96706	15.45948	GW
34	46.96314	15.4461	GW
37	46.95842	15.46766	GW
38	46.82376	15.51175	GW
45	46.80567	15.56993	GW
48	46.84216	15.5745	GW
49	46.97002	15.4106	GW
50	47.17477	15.32404	GW
51	47.17603	15.32611	GW
55	47.23885	15.32486	GW
56	47.24591	15.31498	GW
57	47.18221	14.7553	GW
58	47.17982	14.76385	GW
59	47.13708	14.73891	GW
60	47.15782	14.73747	GW
64	47.21434	14.61545	GW
66	47.20882	14.68559	GW
68	47.19641	14.66202	GW
71	47.20092	14.70705	GW
72	47.18562	14.77964	GW
74	47.19403	14.79267	GW
77	47.122153	13.34591	River
80	47.31095	14.96158	GW
81	47.20431	14.55792	GW
83	47.09098	13.99708	GW
87	47.08298	13.69211	GW
90	47.1208	13.77159	GW
92	47.12973	13.79537	GW

**94**	47.07619	13.9194	GW
**LN 46**	46.7946	15.5306	River
**MUR 1**	46.71456	15.89258	River
**MUR 14**	46.79731	15.58365	River
**MUR 18**	47.07407	15.43471	River
**MUR 23**	47.04703	15.44096	River
**MUR 30**	46.9674	15.48996	River
**MUR 52**	47.17622	15.3261	River
**MUR 67**	47.17228	14.66343	River
**MUR 78**	47.14813	13.37742	River
**MUR 82**	47.20897	14.56002	River
**MUR 85**	47.09272	13.66809	River
**MUR 95**	47.075444	13.92028	River
**PL 65**	47.21119	14.61445	River

Table S2. Methods for photometric measurement of nutrients

**Nutrients**	**Method**
**PO_4^{3-}**	Reduced phosphomolybdic acid method in filtrate as α-phosphomolybdic acid (α-phosphomolybdenum blue), reducing agent ascorbic acid (λ = 890 nm): OENORM DIN EN ISO 6878
**Ps/Pt**	Acidic potassium peroxodisulfate digestion: OENORM DIN EN ISO 6878
**NO_3^-**	UV method in filtrate (λ = 220 nm): APHA 4500-NO3/B
**NO_2^-**	Diazotization method in filtrate as violet red azo dye (λ = 542 nm): DIN EN ISO 26777
**$NH4^+$**	Indophenol blue method in filtrate (λ = 655 nm): DIN 38406-5, OENORM ISO 7150-1
**Ns/Nt**	Alkaline potassium peroxodisulfate digestion (method according to Hansen & Koroleff in: Klaus Grasshoff, Klaus Kremling, Manfred Ehrhardt: Methods of Seawater Analysis. Wiley-VCH 1999, ISBN: 978-3527295890) from filtered/unfiltered sample with subsequent measurement of nitrate according to APHA 4500-NO3/B.
**SiO_4**	Unreduced silicomolybdic acid method in the filtrate as yellow β-silicomolybdic acid (λ = 400 nm): DIN 38405-21

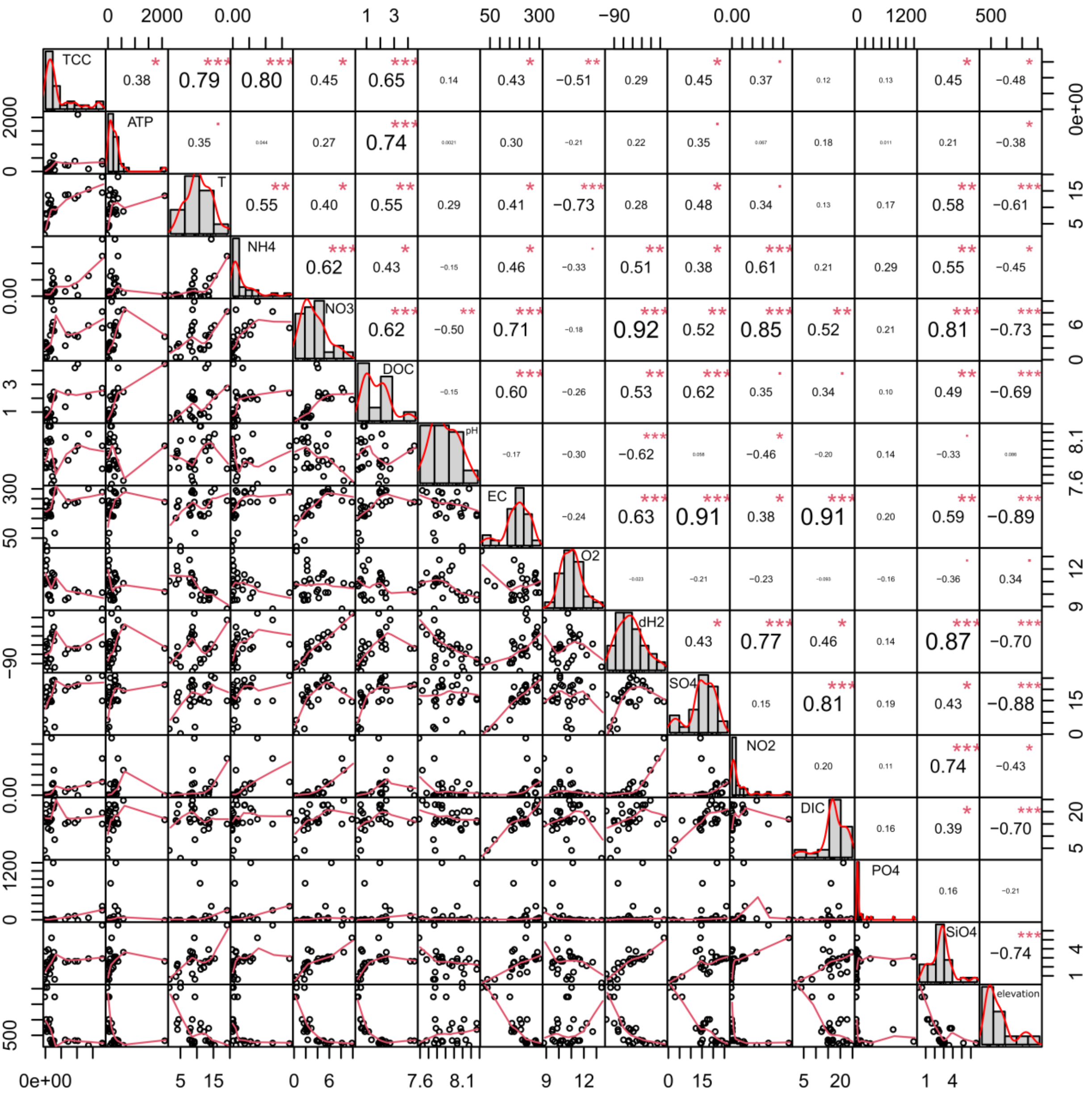

Figure S1. Spearman correlation plot of the most important environmental parameters and microbial activity in Mur River and sidearms. Correlation coefficients are visualized in the upper right part with text size indicating the strength of the correlation and stars represent the level of significance (***p < 0.001, **p < 0.01, *p < 0.05). The lower left part depicts the biplots of each pair of variables. The diagonal shows the distribution of each variable as a histogram. Correlation of variables show that in

surface waters, environmental and microbial variables are much more coupled with than in the groundwater ecosystem.

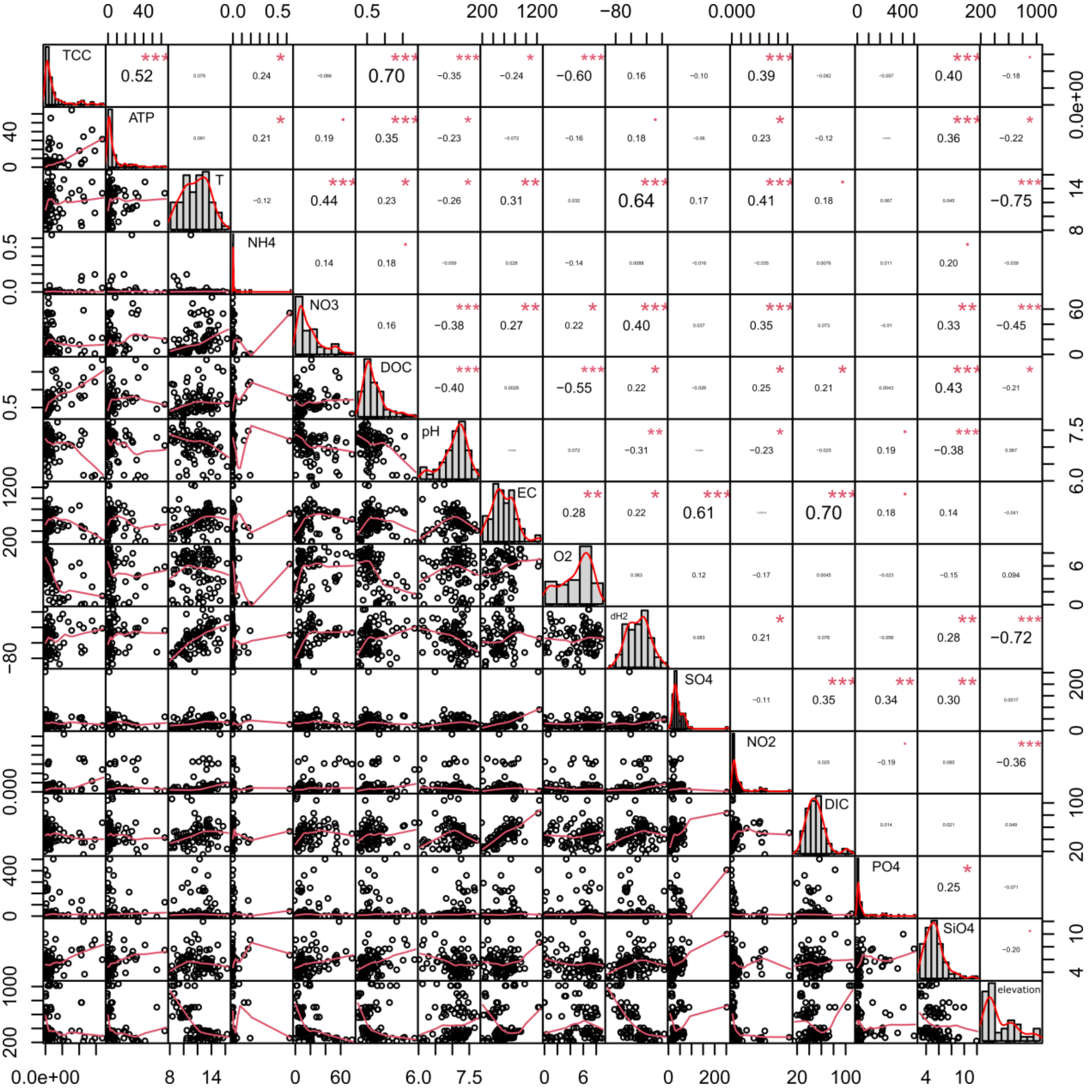

Figure S2. Spearman correlation plot of the most important environmental parameters and microbial activity in groundwater. Oxygen seems to moderately correlate with organic carbon variables (DOM indices, DOC) which in turn have a moderate to strong linear relationship with prokaryotic cell count ($p < .0001$). DOC correlates positively with microbial productivity (prokaryotic cell count and intracellular ATP), and negatively with oxygen ($p < .0001$). Nitrate, elevation, and groundwater temperatures share a moderate to strong linear relationship ($p < .0001$). See Figure S1 on explanation on how to read this graph.

Table S3. Summary of median values of the environmental parameters of Corine Land cover categories and field-, as well as dissolved carbon and nitrate parameters.

Land use	Altitude (m asl)	DO (mg/L)	EC (µS/cm)	pH	T (°C)	NO_3 (mg/L)	DOC (mg/L)	NH4 (mg/L)	NO_2 (mg/L)
**Broad-leaved forest**	238	1.6	565.3	7.0	13.2	21.5	0.99	0.0045	0.0028
**Complex cultivation patterns**	419	7.7	594.3	7.2	11.6	10.8	0.52	0.0019	0.0013
**Continuous urban fabric**	347	5.4	672.0	7.4	14.2	22.9	0.64	0.0077	0.0015
**Discontinuous urban fabric**	367	6.9	548.5	7.2	12.3	9.9	0.51	0.0045	0.0013
**Green urban areas**	376	6.5	844.9	7.0	13.5	18.1	0.60	0.0058	0.0030
**Industrial or commercial units**	359	6.7	698.0	7.3	13.3	18.2	0.52	0.0032	0.0028
**Land principally occupied by agriculture, with significant areas of natural vegetation**	278	5.9	744.5	7.0	12.1	30.4	0.78	0.0032	0.0015
**Mixed forest**	527	0.2	402.3	6.7	9.9	1.2	1.20	0.1601	0.0021
**Non-irrigated arable land**	329	6.4	566.5	7.1	12.3	15.0	0.61	0.0032	0.0010
**Pastures**	874	3.4	281.5	7.4	8.7	4.0	0.33	0.0013	0.0008

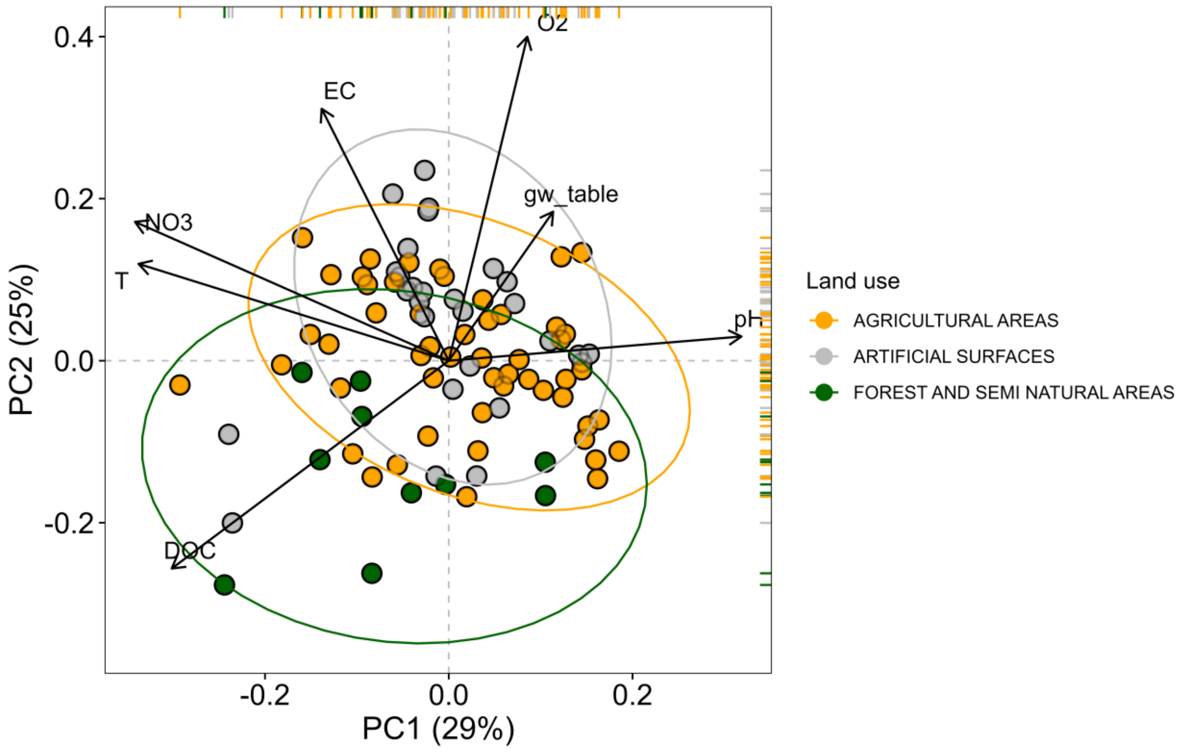

Figure S3. Ordination of z-score transformed environmental variables in groundwater using a principal components analysis (PCA) and samples colored by land use categories.

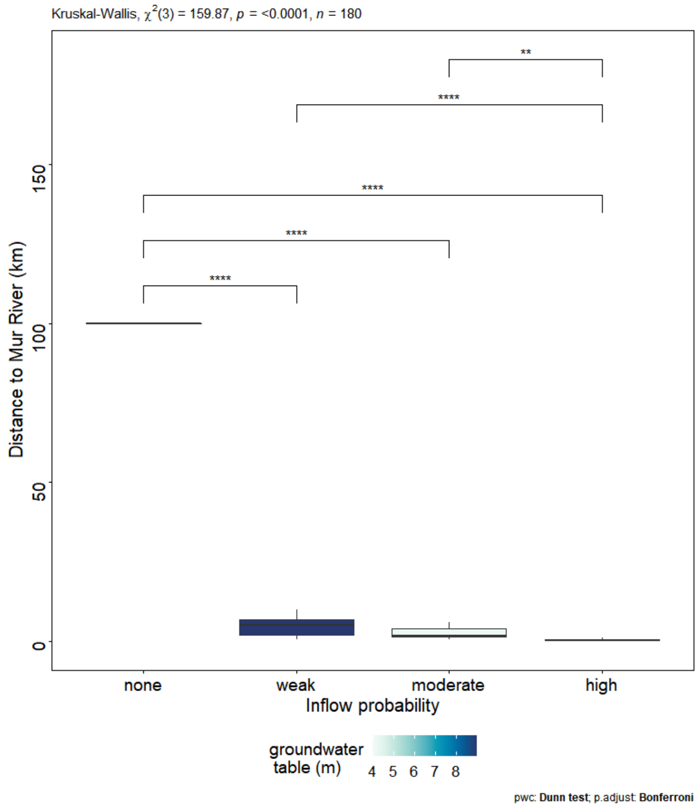

Figure S4. Mur River inflow probability plotted against the distance to the location of the possible inflow, where boxplots are colored in groundwater table depth in m under terrain (Kruskal–Wallis, $\chi^2(3) = 159.87$, $P < 0.0001$).

Table S4. 16S (Bacteria and Archaea) dataset summarizing read statistics, as well as number of ASVs from groundwater.

	spring median # reads	autumn median # reads	tot # reads	# Singleton ASVs	% ASV singletons	ASVs
**DNA**	9484	5455	670083	300	3.9	7621
**RNA**	4406	6260	450872	652	9.2	7128

Table S5. RNA based 16S (Bacteria and Archaea) dataset summarizing read statistics, as well as number of ASVs of groundwater and the Mur River and tributaries.

	tot # reads	# Singleton ASVs	% ASV singletons	ASVs
**Groundwater**	450872	652	9.15	7128
**River**	111338	645	27.49	2346

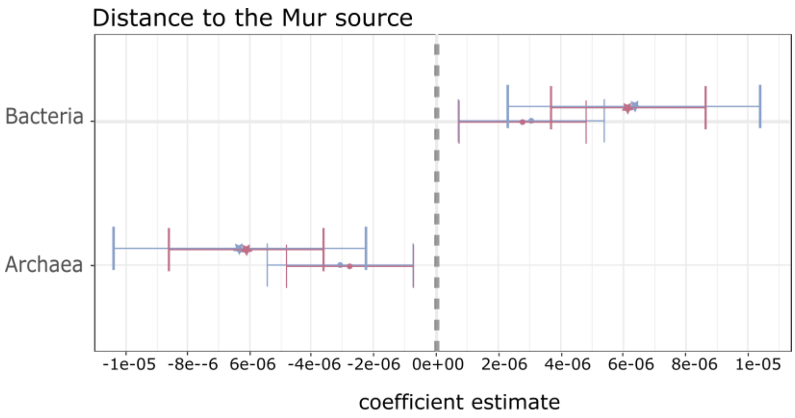

Figure S5. Differentially abundant domains Bacteria and Archaea in the DNA (red) and RNA (blue) fraction between groundwater (circle) and river (star) samples tested against the increasing distance to the Mur source. Model estimation was done using a Wald-test and an FDR cut-off of 0.05.

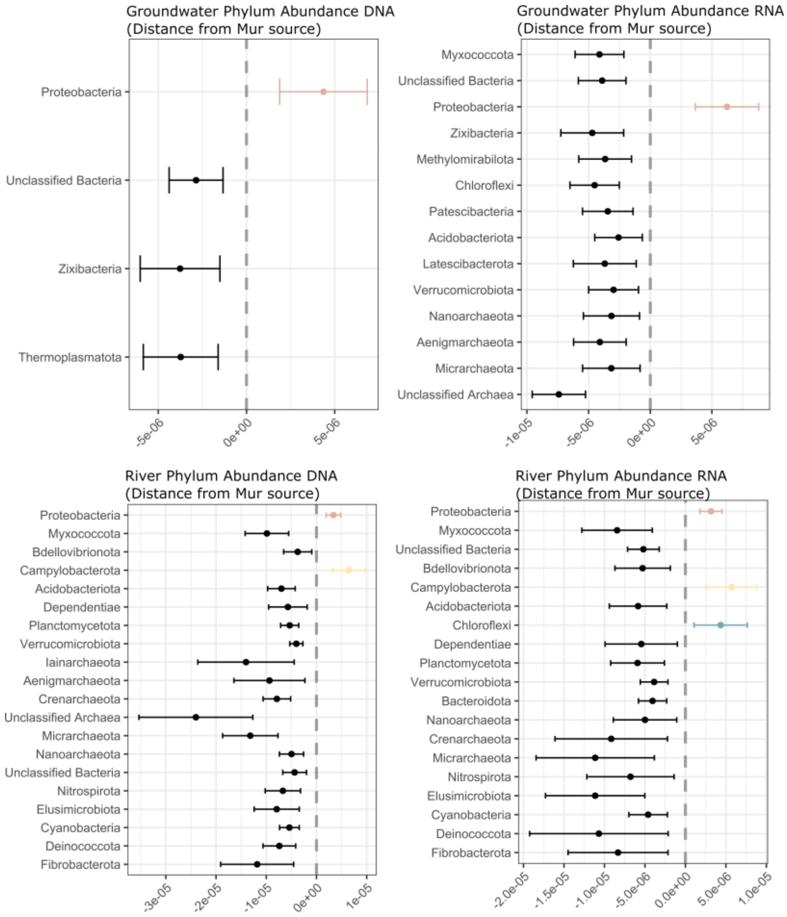

Figure S6. Coefficient estimates of the differentially abundant bacteria and archaea on phylum level in relation to the distance of the Mur source with phyla gaining abundance over the course of the Mur

River transect shown in their respective colors. Model estimation was done using a Wald-test and an FDR cut-off of 0.05.

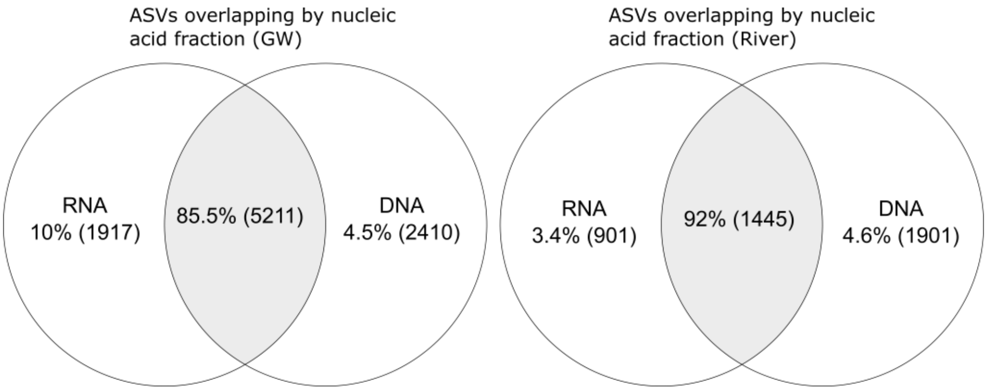

Figure S7. Venn diagram showing the sum of unique, and shared ASVs between the DNA and RNA fraction of the dataset, with overlap weighted by relative abundances, showing shared taxa as counts and percentages as sequences per total sequence number in groundwater (left) and the river (right).

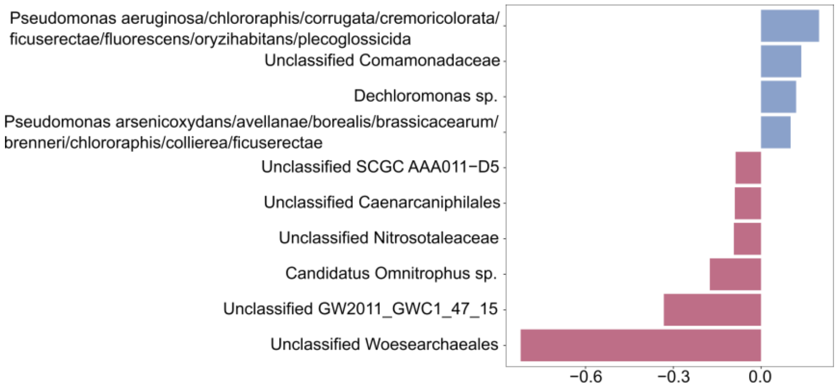

Figure S8. Coefficient estimations of a constrained ordination based on a distance-based redundancy analysis (dbRDA) using Aitchison distances of relative abundance data on species level are plotted in a boxplot to identify the 10 taxa that are most differently abundant in the DNA (red), and RNA (blue) derived communities respectively.

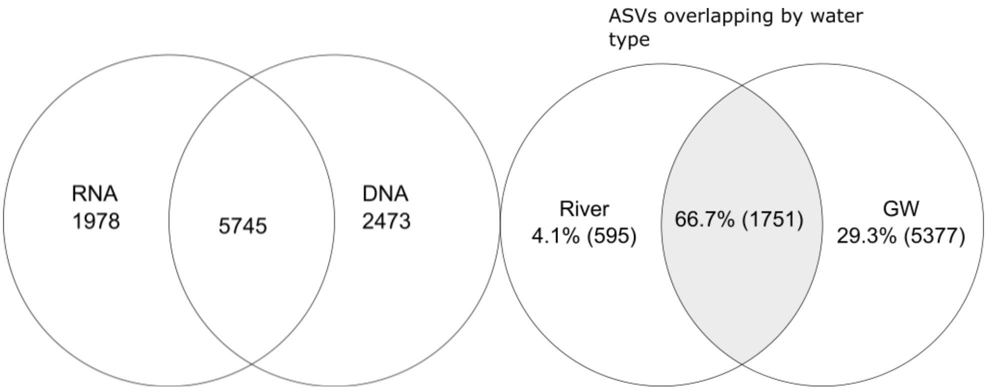

Figure S9. Venn diagram showing the sum of unique, and shared ASVs between the DNA and RNA fraction of the whole dataset (left), as well as between river and groundwater in the RNA fraction (right), with overlap weighted by relative abundances, showing shared taxa as counts and percentages as sequences per total sequence number.

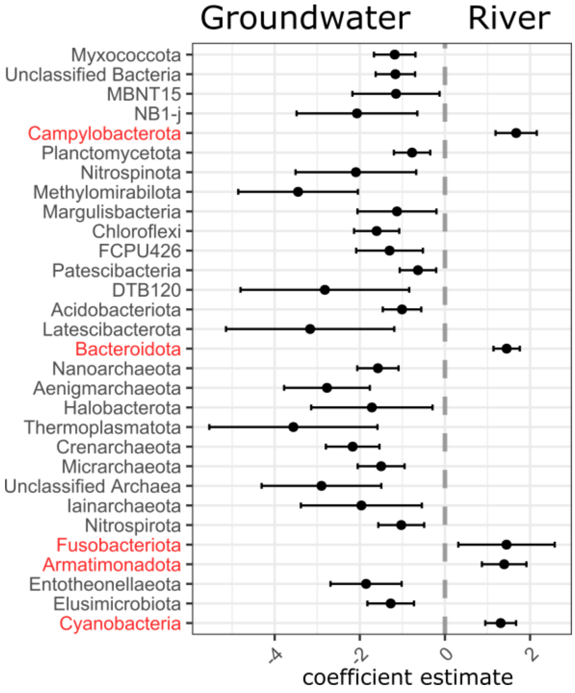

Figure S10. Coefficient estimates of the differentially abundant bacteria and archaea on phylum level in relation to water type with phyla gaining abundance in the surface water shown in red. Model estimation was done with an FDR cut-off of 0.05.

Table S6. Richness statistics estimated with breakaway.

	Total estimated richness	SE	Observed richness	% Unseen species
**Groundwater**				
Breakaway (no singletons)	144913	5619576	31762	78
Breakaway frequency cutoff 10	331167	82109	58740	82
**River**				
Breakaway (no singletons)	18487	18704	6582	64
Breakaway frequency cutoff 10	35246	2423	11276	68

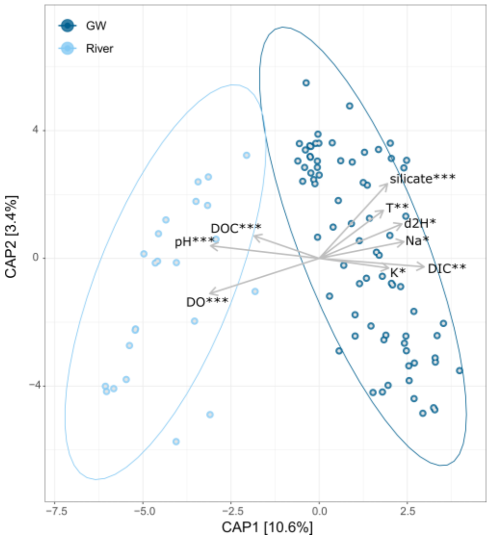

Figure S11. Ordination of species turnover with a constrained analysis of principal coordinates (CAP) based on the clr transformed abundances between groundwater and the river in association with respective nutrients, as well as physico chemical parameters (***p < 0.001, **p < 0.01, *p < 0.05).

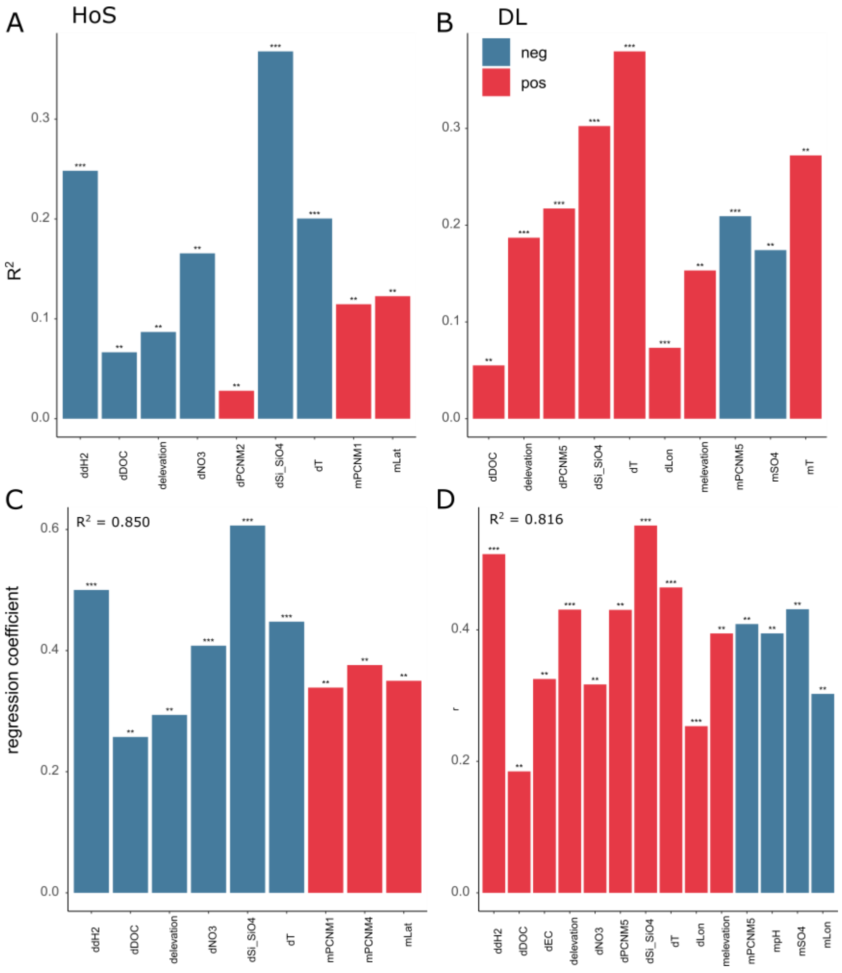

Figure S12. Correlations of environmental variables showing factors with significant correlations for HoS (A) and DL (B) based on a Mantel analysis. Multiple regression on distance matrix (MRM) for HoS (C) and DL (D) with regression coefficient on the y-axis and R2 shown based on the model from the Mantel test, which was based on log-transformed data. Negative correlations are colored in blue, and

positive in red. Correlation was based on the difference (d) or the mean (m) of the variables between each pair of samples (***p < 0.01; **p < 0.05).

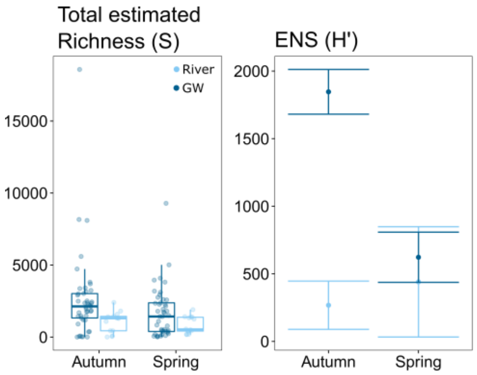

Figure S13. Diversity metrics of groundwater (dark blue) and river (light blue) total (DNA derived) prokaryotic communities between spring and autumn. Richness is shown in form of a boxplot showing median values as midline, first and third quantiles as edges, and whiskers representing the max/min values. Shannon diversity is shown as effective species with error bars showing the mean estimates from covariate-wise estimations, as well as standard errors of the mean.

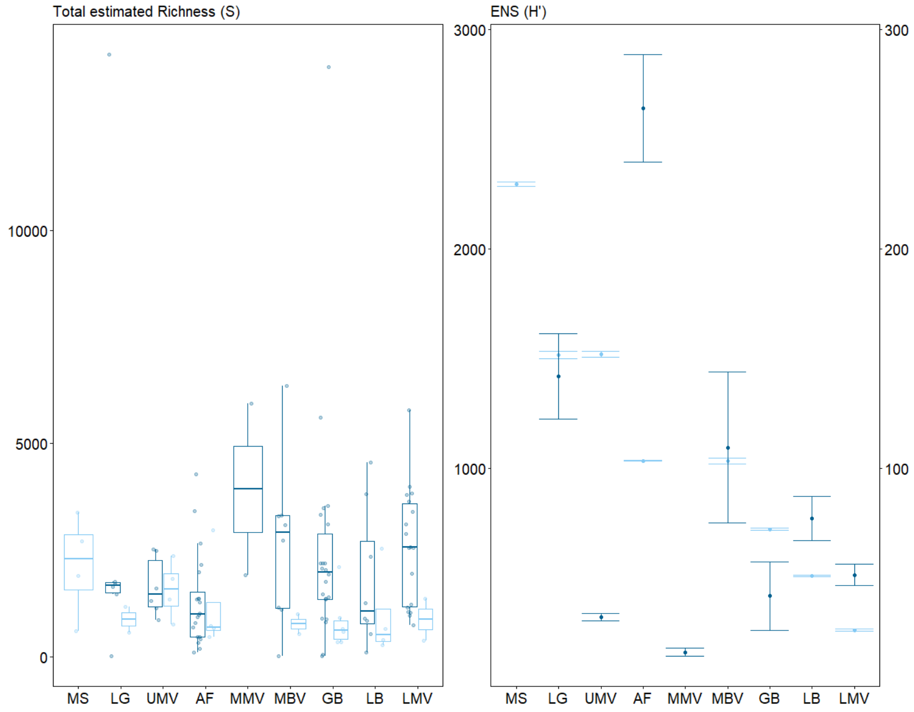

Figure S14. Diversity metrics of groundwater and river total (DNA derived) prokaryotic communities between regions. Richness is shown in form of a boxplot showing median values as midline, first and third quantiles as edges, and whiskers representing the max/min values. Shannon diversity is shown as

effective species with error bars showing the mean estimates from covariate-wise estimations, as well as standard errors of the mean (groundwater = dark blue, river = light blue).

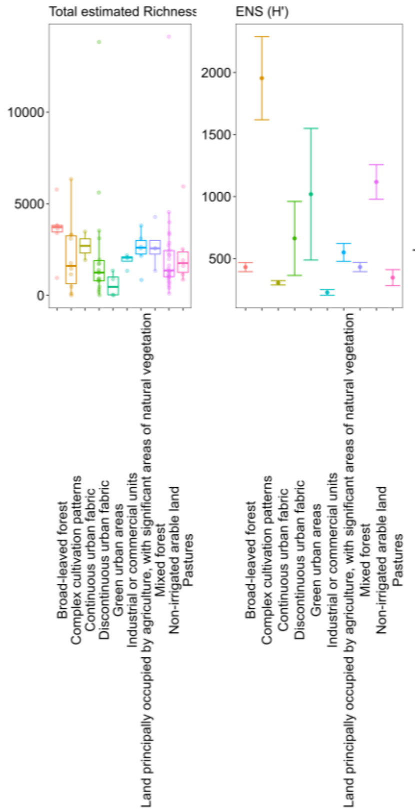

Figure S15. Diversity metrics of groundwater total prokaryotic communities between land use categories. Richness is shown in form of a boxplot showing median values as midline, first and third quantiles as edges, and whiskers representing the max/min values. Shannon diversity is shown as effective species with error bars showing the mean estimates from covariate-wise estimations, as well as standard errors of the mean.
